# Ecological contributions to body shape evolution in salamanders of the genus *Eurycea* (Plethodontidae)

**DOI:** 10.1371/journal.pone.0216754

**Published:** 2019-05-15

**Authors:** Hilary A. Edgington, Douglas R. Taylor

**Affiliations:** 1 Department of Entomology, The Ohio State University, Wooster, OH, United States of America; 2 Department of Biology, University of Virginia, Charlottesville, VA, United States of America; Universitat Trier, GERMANY

## Abstract

**Background:**

Body shape can be both a consequence and cause of a species’ evolution and ecology. There are many examples of phenotypes associated with specific ecological niches, likely as a result of specific selective regimes. A classic example of this is the phenotypic change associated with colonization of caves, including body and limb elongation. However, studies explicitly testing for differences in body shape between cave-dwelling and non-cave-dwelling lineages have been limited and so the role of the cave environment in determining morphological characteristics is still not completely understood. Here we examine variation in body shape among 405 individuals representing 20 species in the salamander genus *Eurycea* (Plethodontidae) and select outgroups exhibiting great diversity in morphology, ecological niche, and life history.

**Results:**

After analyzing morphometric data in a phylogenetic context using phylogenetic MANOVA and examination of the phylomorphospace, we found significant differences in body shape among cave-dwelling and non-cave-dwelling species and between aquatic and terrestrial species. Notably, limb elongation and reduced body and tail size characterized cave-dwelling species. Terrestrial species also exhibited elongation of the limbs and digits. We also observed differences in shape variance among paedomorphic and biphasic species. Our results suggest that the functional limitations imposed by habitat and life history played a key role in the evolution of body shape in this group in the context of their phylogenetic history.

## Introduction

Body shape is a key part of morphological variation among vertebrates, with impacts on function and ecology [1–2]. Variation in shape may be a result of environmental effects, structural or functional constraints, adaptive differentiation, or shared phylogenetic history [3–5]. There are many examples of body shape divergence that have been attributed completely to adaptation to ecological circumstance [6–8], differences of function (e.g. the use of limbs for running across open ground versus clinging to rocky outcrops) [9–12], or a combination of the two [13–14]. Often, patterns of morphological variation are shaped by shared phylogenetic history [15–16], which may influence variation in function or behavior [17]. Understanding the causes of variation in body shape is important for understanding how it may impact a species’ evolutionary trajectory: for example, increased fitness from the evolution of a certain body shape may prevent divergence from that shape, whereas similarity due to shared evolutionary history may not limit future changes in morphology.

Elongation of body or limb is a specific axis of morphological variation that has long been included in a suite of traits associated with cave-dwelling species [18–24]. Cave-dwelling taxa are of particular interest because of their dramatic morphological and physiological changes, the simplicity of the selection regime within the cave habitat, and the resulting parallel evolution of cave-associated traits [25–26]. These traits, known collectively as troglomorphy [19], include other features such as regression of eyes, depigmentation, enhanced extra-optic sensory structures, and reduced metabolism [26]. Troglomorphic traits result from both a relaxation of selection pressures formerly experienced in the ancestral surface habitats, and as a result of directional selection experienced within the cave environment [27]. Though most cases of troglomorphic elongation have been studied in invertebrates due to their relative abundance (e.g. 25,19), studies of cave vertebrates, and salamanders in particular, also associate elongation with cave-dwelling [20,28–29].

In this study we examined the impact of habitat and life history on body shape and size by comparing morphological measurements among (1) aquatic and terrestrial and (2) cave-restricted and non-cave-restricted species in the salamander genus *Eurycea*. This group is well suited to studies of phenotypic evolution because of the extensive morphological and ecological variation represented therein: it exhibited exceptionally high rates of both size and shape evolution when compared with other plethodontids [30], and inhabits most known ecological niches available to salamanders. In addition, the independent colonization of caves and of aquatic habitats by multiple lineages of *Eurycea* [31] presents a natural experiment in potential ecological roles in morphological variation.

This work addresses a number of issues with our current knowledge of the evolution of body elongation as it relates to habitat occupancy: First, we analyzed the relationship between habitat and trait evolution in the context of phylogenetic relatedness. Though it is important to consider trait evolution in the context of patterns of relatedness in order to avoid bias [32–33], troglomorphic elongation has not been assessed in this group using phylogenetically based statistical methods to our knowledge. Past comparisons that find differences in shape among cave-dwelling and non-cave-dwelling populations [20,28–29] have focused mainly on the Texas clade of cave-dwelling and non-cave dwelling *Eurycea*, which are entirely aquatic, and none have included phylogenetic context. By studying a broader taxonomic, ecological and morphological sampling and analyzing trait differences in a phylogenetic context, we were able to compare species in a variety of habitats, providing a greater insight into the relationship between ecology, phylogeny, and morphology.

## Materials and methods

### Morphometric data collection

In December 2013 and February 2015 we took photographs of 405 preserved specimens representing 20 species of *Eurycea* in the herpetology collections of the American Museum of Natural History (New York City, New York) and the Smithsonian Institution National Museum of Natural History (Washington, D.C.). Photographs included three angles (dorsal, ventral, and lateral views), and a size standard. Because sexual size dimorphism is minor relative to individual size variance in salamanders [34], we did not attempt to collect data on sex from these specimens. We measured nine morphometric traits from these photographs using the image processing software ImageJ (NIH). These traits include: head width, forelimb length, forelimb width, body width at its widest between the forelimbs and hindlimbs, hindlimb length, hindlimb width, the length of the fourth back digit, tail length, and snout-vent length (SVL). Where the tail tip was missing or undergoing regrowth we did not measure tail length and left it as missing data. One person performed all of the digital processing to avoid among-researcher error in measurement (data can be found in S1 Table). Using information from [35], [36], and [34], we recorded whether each species is aquatic or non-aquatic and restricted to caves or not, and recorded whether each species exhibits obligate paedomorphosis, facultative paedomorphosis, or obligate metamorphosis (Table 1).

**Table 1:** Species included in this study, together with their documented primary habitat and whether they exhibit paedomorphosis (N = no; F = facultatively; Y = obligately).

Species	Cave	Aquatic	Paedomorphic
Eurycea aquatica	Non-cave	Aquatic	N
Eurycea bislineata	Non-cave	Aquatic	N
Eurycea cirrigera	Non-cave	Aquatic	N
Eurycea guttolineata	Non-cave	Terrestrial	N
Eurycea junaluska	Non-cave	Terrestrial	N
Eurycea latitans	Cave	Aquatic	Y
Eurycea longicauda longicauda	Non-cave	Terrestrial	N
Eurycea longicauda melanopleura	Non-cave	Terrestrial	N
Eurycea lucifuga	Cave	Terrestrial	N
Eurycea multiplicata	Non-cave	Aquatic	F
Eurycea nana	Non-cave	Aquatic	Y
Eurycea neotenes	Non-cave	Aquatic	Y
Eurycea pterophila	Cave	Aquatic	Y
Eurycea quadridigitata	Non-cave	Terrestrial	N
Eurycea rathbuni	Cave	Aquatic	Y
Eurycea spelaea	Cave	Terrestrial	N
Eurycea tridentifera	Cave	Aquatic	Y
Eurycea tynerensis	Non-cave	Aquatic	Y
Eurycea wallacei	Cave	Aquatic	Y
Eurycea wilderae	Non-cave	Terrestrial	N
Gyrinophilus porphyriticus	Cave	Terrestrial	N
Hydromantes brunus	Non-cave	Terrestrial	N
Hydromantes genei	Cave	Terrestrial	N
Hydromantes italicus	Cave	Terrestrial	N
Hydromantes platycephalus	Non-cave	Terrestrial	N
Proteus anguinus	Cave	Aquatic	Y

### Phylogenetic reconstruction

**Fig 1:**
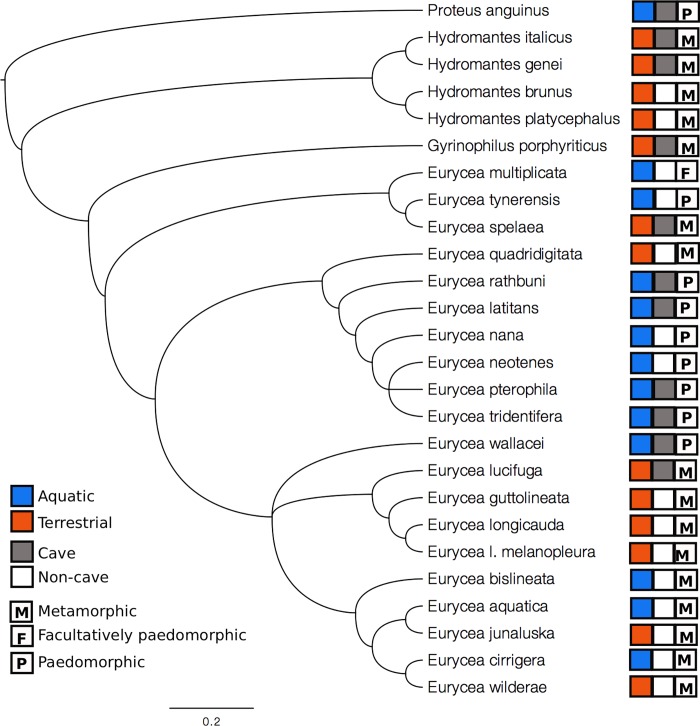
Phylogenetic tree of the *Eurycea* and outgroups. Tree was obtained from [37]. Phylogenetic history was reconstructed using three mitochondrial (*Co1*, *Cytb*, and *ND2*) and four nuclear (*BDNF*, *Pomc*, *RAG1*, and *Slc8a3*) genes using the Bayesian software BEAST 2.4. Annotations represent habitat and life history characteristics of each species.

Phylogenetic variance-covariance among species was estimated using a previously published phylogenetic reconstruction [37]. We obtained results of a BEAST species tree reconstruction including all of our sampled species, estimated the consensus tree which included clades represented in greater than 90% of trees and computed branch lengths using the package *ape* v5.2 [38], and estimated the variance-covariance matrix from this tree using the package *geiger* v2.0.6 [39]. This consensus tree was visualized using FigTree v1.4.0 (Fig 1; [40]).

### Statistical analysis

All statistical analyses were performed using R v3.1.2 [41] interfaced through RStudio v0.98.1091 (RStudio, Inc.). We transformed our morphological measurements using log-shape ratios [42] and first tested for evidence of phylogenetic signal influencing these morphological traits using the R package *phylocurve* v2.0.9 [43]. Since relatedness among species may impinge on the independence of these data, we analyzed them in a phylogenetic context. We tested for significant differences in body shape measurements using a permutational phylogenetic MANOVA with the R package *RRPP* v0.4.0 [44,45]. We used the Pillai statistic to test for associations between the set of log-shape ratios computed for the measured body shape variables and life history/habitat, including as independent variables cave/non-cave, aquatic/terrestrial, and paedomorphic/facultatively paedomorphic/biphasic. Data were visualized using the R package *ggplot2* [46].

### Phylomorphospace plot

We also performed a principal component analysis (PCA) on the transformed body shape measurements using the function prcomp() in the R package *stats* [41], centering and scaling the data. Studying principal components, a common strategy in morphometric analyses [37,47–50], allows us to analyze statistically uncorrelated variables, control for the effects of size and individual variation, and reduce the number of variables. We first interrogated the first three principal components for phylogenetic signal using the function phylosig() in the *phytools* package [51]. In order to support our statistical models with a visual interpretation of these data we produced a phylomorphospace plot of the first three principal components. This projects a phylogenetic tree into two-dimensional morphological space, which provides an intuitive way to identify clustering of discrete traits and convergent evolution. We used the function phylomorphospace() in the R package *phytools* [51].

## Results

### Phylogenetic MANOVA

Comparison with a null star phylogeny revealed that body shape exhibits significant phylogenetic signal (K = 0.268, p < 0.0001; S1 Fig). Phylogenetic MANOVA indicates significant impacts of habitat but not life history on body shape in this group. Body shape was significantly different between cave and non-cave species, as well as between aquatic and terrestrial species (Table 2A). Differences among obligately and facultatively paedomorphic and biphasic species were trending towards significance. Generally, non-cave species exhibited increased tail length, narrowing of the head, and limb reduction compared with cave species. Terrestrial species exhibited limb and digit elongation relative to body size when compared with aquatic species. Though differences were not statistically significant, paedomorphic species tended to have wider heads and more elongated bodies and limbs than biphasic species (Table 2B).

**Table 2:** Phylogenetic MANOVA reveals significant differences in log-shape-ratio transformed shape measurements between cave and non-cave species, and between aquatic and terrestrial species. a) MANOVA test statistics comparing measurements among habitat and life history categories. b) Regression coefficients show the direction and effect size of each relationship.

**a.**	**df**	**Pillai**	**Z**	**Pr(>Pillai)**					
Cave	1	0.845	2.465	**0.001**					
Aquatic	1	0.905	2.575	**0.001**					
Paedomorphic	2	0.867	1.405	0.062					
Full Model	4	1.685	2.436	**0.004**					
**b.**	**Head** **Width**	**Forelimb** **Length**	**Forelimb** **Width**	**Body** **Width**	**Hindlimb** **Length**	**Hindlimb** **Width**	**Digit** **Length**	**Tail** **Length**	**SVL**
Non-cave vs. Cave	-0.035	-0.023	-0.048	0.017	0.003	-0.014	-0.039	0.115	0.025
Terrestrial vs. Aquatic	-0.016	0.046	-0.016	-0.019	0.032	-0.014	0.017	-0.006	-0.024
Paedomorphosis (Linear)	0.038	0.041	-0.064	-0.004	0.014	-0.093	-0.001	0.012	0.057
Paedomorphosis (Quadratic)	0.008	0.013	-0.042	-0.010	0.008	-0.026	0.040	0.019	-0.010

### Principal component analysis

**Fig 2:**
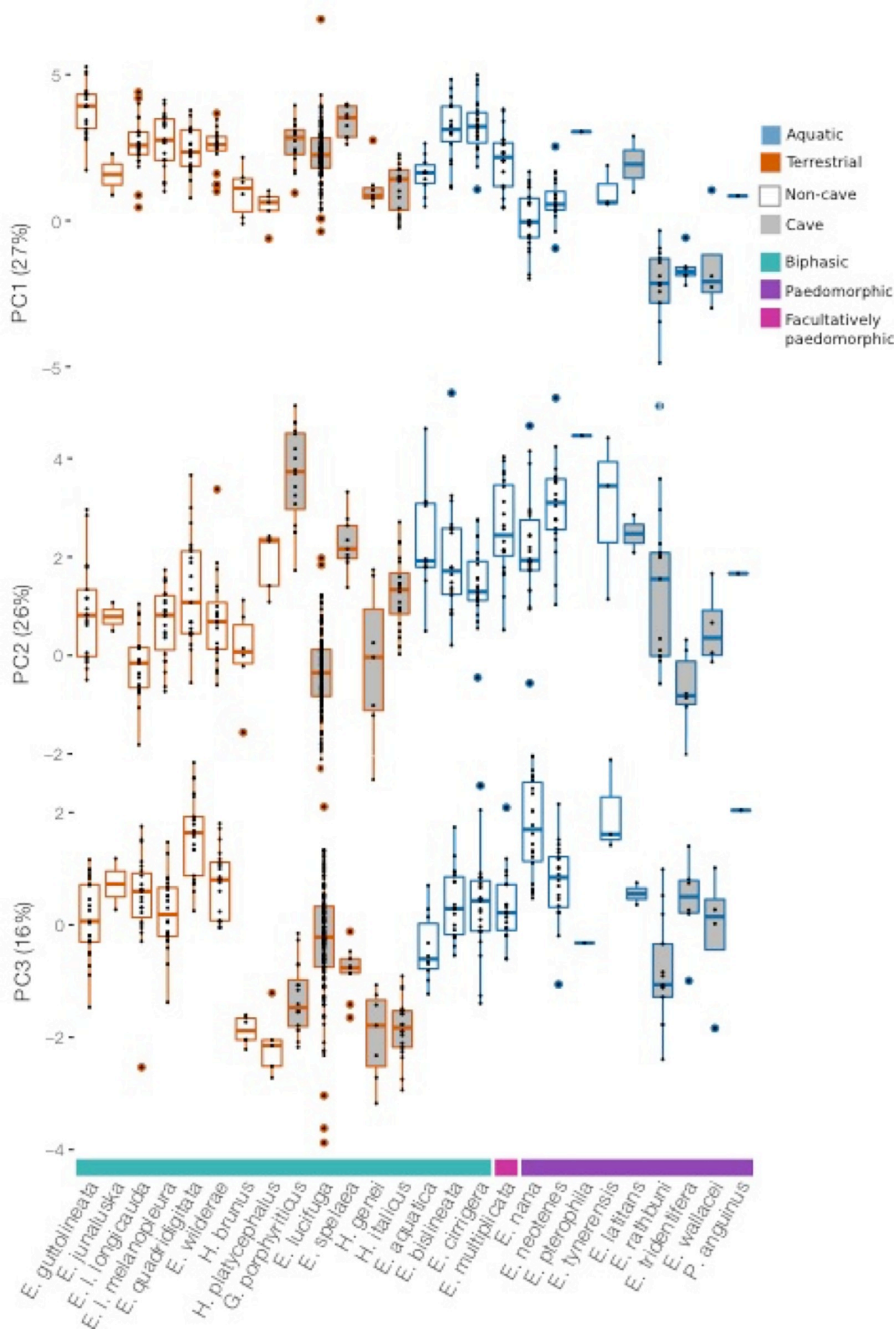
Comparisons of the first three principal components among species. PC1 represents wider, shorter limbs, longer torsos relative to tail length, and narrower heads. PC2 represents wider heads and bodies and longer tails relative to SVL, and shorter limbs and digits. PC3 is representative of an increase in body and tail length, and decreases in limb size and head width.

Principal component analysis (PCA) was performed on transformed morphometric data from 20 species of *Eurycea* (Fig 2). The first three principal components accounted for 69% of the cumulative variance (Table 3). PC1, which accounted for 27% of the total variance, represented reduced head width and tail length, and shorter, fatter limbs relative to SVL. PC2 (26% of total variance) represented increased head and body width, longer tails, and shorter limbs relative to SVL. PC3 (16% of total variance) represented general elongation- an increase in SVL and tail length relative to decreased head and body width, and reduction of limb size. To ensure our interpretations of the directionality of the principal components was correct we visualized the relationships between each principal component and its strongest loading trait (S1 Fig). We found that phylogenetic relatedness significantly influences the distribution of principal component 3 (Table 4).

**Table 3:** Principal Component reductions of body shape. Log-shape ratios were used to perform a principal components analysis, after centering and scaling the data. Eigenvectors are reported here, together with the proportion of variance assigned to each component. The first three principal components were used in phylomorphospace visualizations.

	PC1	PC2	PC3	PC4	PC5	PC6	PC7	PC8	PC9
Head Width	-0.454	0.320	-0.198	0.087	-0.076	0.251	0.367	-0.607	0.269
Forelimb Length	-0.411	-0.338	-0.246	-0.192	-0.277	0.306	0.171	0.577	0.295
Forelimb Width	0.366	0.174	-0.481	-0.011	-0.610	-0.278	-0.240	-0.076	0.304
Body Width	-0.054	0.555	0.012	0.141	0.248	0.399	-0.549	0.271	0.270
Hindlimb Length	-0.379	-0.349	-0.116	-0.323	0.289	-0.328	-0.528	-0.289	0.251
Hindlimb Width	0.423	0.004	-0.422	-0.214	0.601	-0.005	0.360	0.074	0.317
Digit Length	0.005	-0.325	0.076	0.822	0.103	-0.169	0.030	0.037	0.414
SVL	0.326	-0.161	0.608	-0.299	-0.169	0.266	0.019	-0.196	0.521
Tail Length	-0.244	0.438	0.321	-0.141	0.008	-0.632	0.259	0.300	0.262
Proportion of variance:	0.270	0.258	0.158	0.119	0.064	0.052	0.045	0.033	0.000

**Table 4:** Results of tests for significant phylogenetic signal using the K statistic in each of the four first principal components. Σ^2^ estimates the rate of evolution for each PC. Phylogenetic relatedness was included in linear models for those PCs with significant phylogenetic signal.

	K	p	Σ ^2^
**PC1**	0.242	0.136	37.186
**PC2**	0.122	0.628	46.548
**PC3**	1.1	**0.001**	9.586

### Visualizations of morphological space

**Fig 3:**
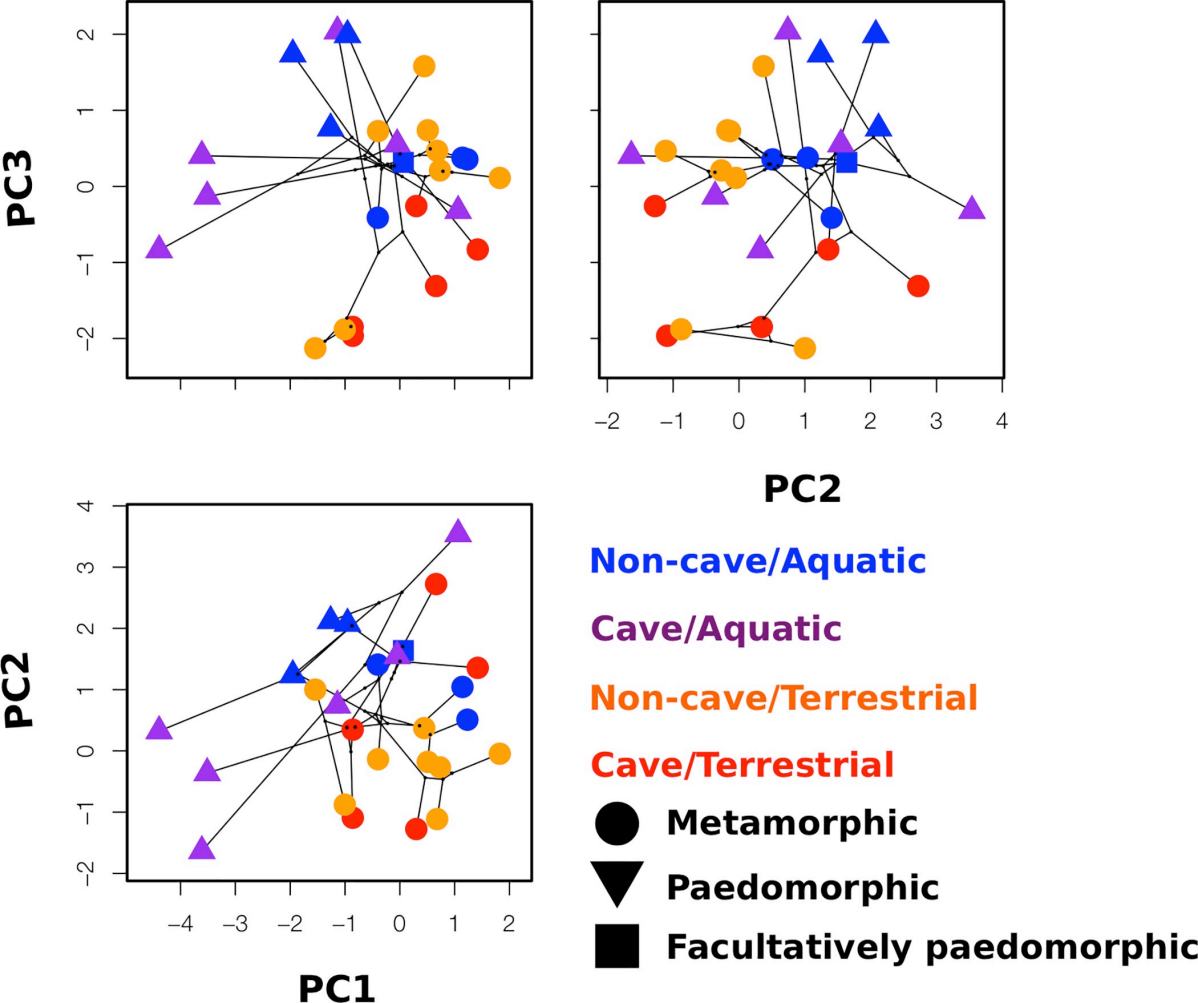
Phylomorphospaces depicting the phylogenetic relatedness among these species in the space defined by the first three principal components, and differentiated by both habitat and life history. Clustering similar to glmm results can be observed: segregation between paedomorphic and metamorphic species along the PC1 axes, and between both paedomorphic and metamorphic species and cave and non-cave species along the PC3 axes.

**Fig 4:**
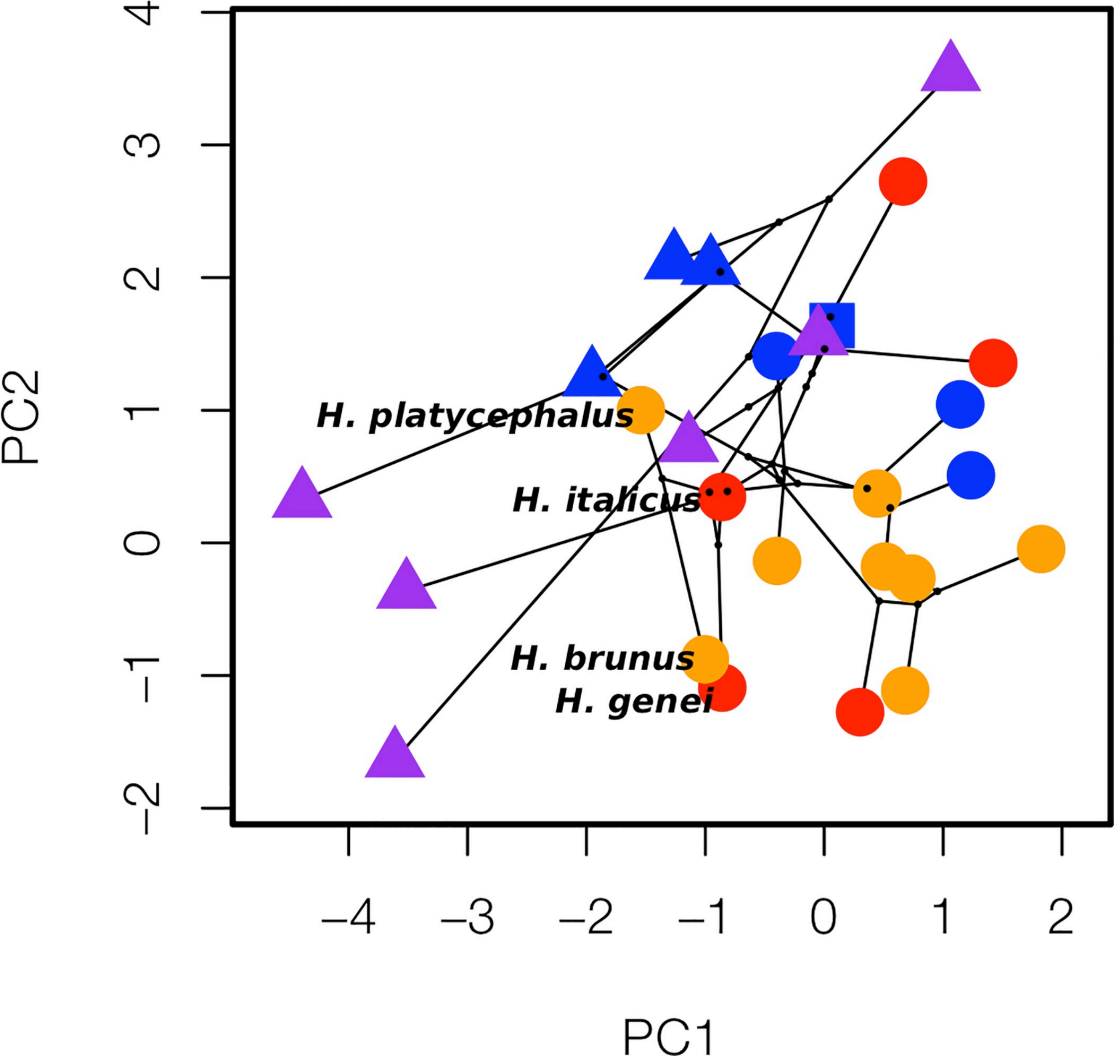
Clustering among the Hydromantes in contrast to their relatedness and shared ranges and habitats suggests a complex evolutionary history underlies morphology in this group.

We used phylomorphospace plots to visualize the variance in each of the principal components exhibiting significant associations with habitat in our analyses (Fig 3). We see not only the segregation of biphasic/paedomorphic species along the PC1 and PC3 axes, as uncovered in the glmm results, and the segregation of cave/non-cave species along the PC3 axes, but also some interesting patterns not observed with our linear models. We observed apparent differences in variance among groups in our phylomorphospace plots, which were confirmed by the results of multiple Breusch Pagan tests for heteroscedasticity (Table 5). Most consistently, paedomorphic species had significantly more variance in their morphology than biphasic species. This is most visually apparent in the comparison of PC1 and PC2 (Fig 2), where paedomorphic species have markedly long branches and span the entire morphospace. Aquatic and terrestrial species also differed in the variance they exhibit, but inconsistently. We also see interesting morphological clustering among unrelated species, as the case of the Hydromantes in the PC1/PC2 comparison: clustering of *Hydromantes brunus* with *H*. *genei* and *H*. *italicus* with *H*. *platycephalus* (Fig 4) indicates shared morphological features despite closer relationships, sympatry, and shared ecological requirements of *H*. *brunus* with *H*. *platycephalus* and *H*. *genei* with *H*. *italicus*.

**Table 5:** Tests of heteroscasdicity among principal components. We performed Brausch Pagan tests for heteroscedasticity on linear models including each principal components and our independent variables in order to examine variance among groups. Results, reported as X^2^(p value), indicate different ecological and life history groups exhibit more variance in their morphologies depending on the trait. Significant results, following a Holm-Bonferroni correction for multiple comparisons, are in bold.

	PC1	PC2	PC3
Cave/Non-cave	1.495(0.443)	**27.991(<0.001)**	1.164(0.561)
Aquatic/Terrestrial	**11.698(0.002)**	1.423(0.466)	**6.458(0.044)**
Paedomorphosis	**23.572(<0.001)**	0.335(0.563)	0.544(0.461)

## Discussion

Our goal in these analyses was to examine the impact of habitat and life history on body shape among 26 species of Plethodontid salamanders. Our results indicate that habitat has significantly shaped the morphology of these species, while life history may have also played a role. We also found that examination of some morphological traits requires consideration of the underlying phylogenetic relationships. Specifically, we found that cave species do indeed tend to have more elongated limbs compared with non-cave species, together with shorter tails. We also found that terrestrial species exhibit elongation of limbs compared with aquatic species, and that paedomorphic species tended towards elongation of limbs and torsos and wider heads compared with biphasic species. These results at a broad taxonomic scale, though only marginally significant, reflect recent findings that within *Eurycea tynerensis*, a facultatively paedomorphic species found in the Ozark Plateau, paedomorphic populations have an increased number of vertebrae compared with biphasic populations [52]. Additionally, it was previously shown in the *Eurycea* that rates of diversification in the vertebral column was found to be dramatically greater among paedomorphic species than biphasic species, which the authors attribute to the imposition of conflicting selective constraints across ontological stages [37]. The general elongation of paedomorphic species together with the limb elongation in cave species we also observe here may contribute to the perception that cave-dwelling species tend toward general elongation [18–24], as the majority of well-known cave obligates are paedomorphic.

Variation in morphology arises through many different mechanisms including environmental influences, structural or functional constraints, or shared evolutionary history [3–5]. Some taxa show distinct differences among populations due primarily to ecological differences [10,15,53], which may be driven primarily by functional differences in how traits benefit organisms in those habitats (e.g., climbing requires different adaptations than swimming or burrowing [54]). Other taxa exhibit a combination of ecology-driven and phylogeny-driven variation among lineages [54–55]. Somewhat surprising among our observations is the minor role that phylogenetic signal plays in the body shape traits we examine when variation is reduced using principal components- we found significant phylogenetic signal in only principal component three, which represents a mere 16% of variance in these morphological traits. The first two principal components, representing 53% of total variance, exhibited no significant phylogenetic signal and this may suggest that much of body shape has evolved in these species as a result of their habitat and life history, and independent of evolutionary origin. The results of our tests for phylogenetic signal, phylogenetic MANOVA, and phylomorphospace visualizations indicate that variation body size and shape in *Eurycea* and similar Plethodontids is driven by a complex interaction of ecology, life history, and phylogeny.

The shape differences we observe between cave-dwelling and non-cave-dwelling species allow us to make hypotheses about the functional implications of elongation in this group. While the literature leads us to expect to observe elongation of cave species, we find here that they exhibit general reduction of the torso and head and shortened tails. While this is unexpected, it could be hypothesized that the energetic costs of the cave habitat, in which organisms face cold temperatures, high humidity, and a paucity of resources [25–26] contributes to the general body and tail reduction of cave species in this group. Energetic costs of elongation have been observed in other species [56], and in the relatively extreme cave environment those costs may have resulted in adaptive morphological change toward a more metabolically efficient shape. The increase in limb length in contrast to the reduction in body and tail size exhibited by cave-dwelling species can be hypothesized to result from ambulatory requirements of caves. Limb length has been correlated with running speed in a large study of mammalian species, though it was predicted that reducing costs of locomotion may be a stronger influence on the evolution of limb form than potential speed [57], and has also been found to differ among arboreal and terrestrial species in studies of lizards, which is hypothesized to represent trade-offs between traits benefiting running and climbing [11,58,59]. The need for salamanders to climb rocky walls and cling to crevices may drive the evolution of long limbs in cave-dwellers that we see here.

Together our results suggest that the evolution of body shape is largely influenced by life history and habitat in these Plethodontids, along with their phylogenetic relatedness to a lesser degree. Similar evolution due to ecological niche occupancy has been found in other systems, such as the convergent reductions in bone size in freshwater threespine sticklebacks [8] and repeated elongation within families of reef fish [60]. As many of these species are of conservation concern due to their endemism in miniscule ranges, it is important to note that habitat preservation in these cases is critical not only for the maintenance of species diversity but also morphological diversity.

## Supporting information

S1 Fig Correlations between principal components and shape measurements. (PDF)

S1 Table Raw measurement data for all traits for each individual in this study. (DOCX)
