# Supplementary material for: Ecological contributions to body shape evolution in salamanders of the genus Eurycea (Plethodontidae)
Source: PLoS One. 2019 May 15;14(5):e0216754. doi: 10.1371/journal.pone.0216754 (PMC6519905; doi:10.1371/journal.pone.0216754)

Supplementary Figure S1. To ensure we interpreted the principal components analysis correctly we visualized a comparison of each PC and its most prominent morphological feature.

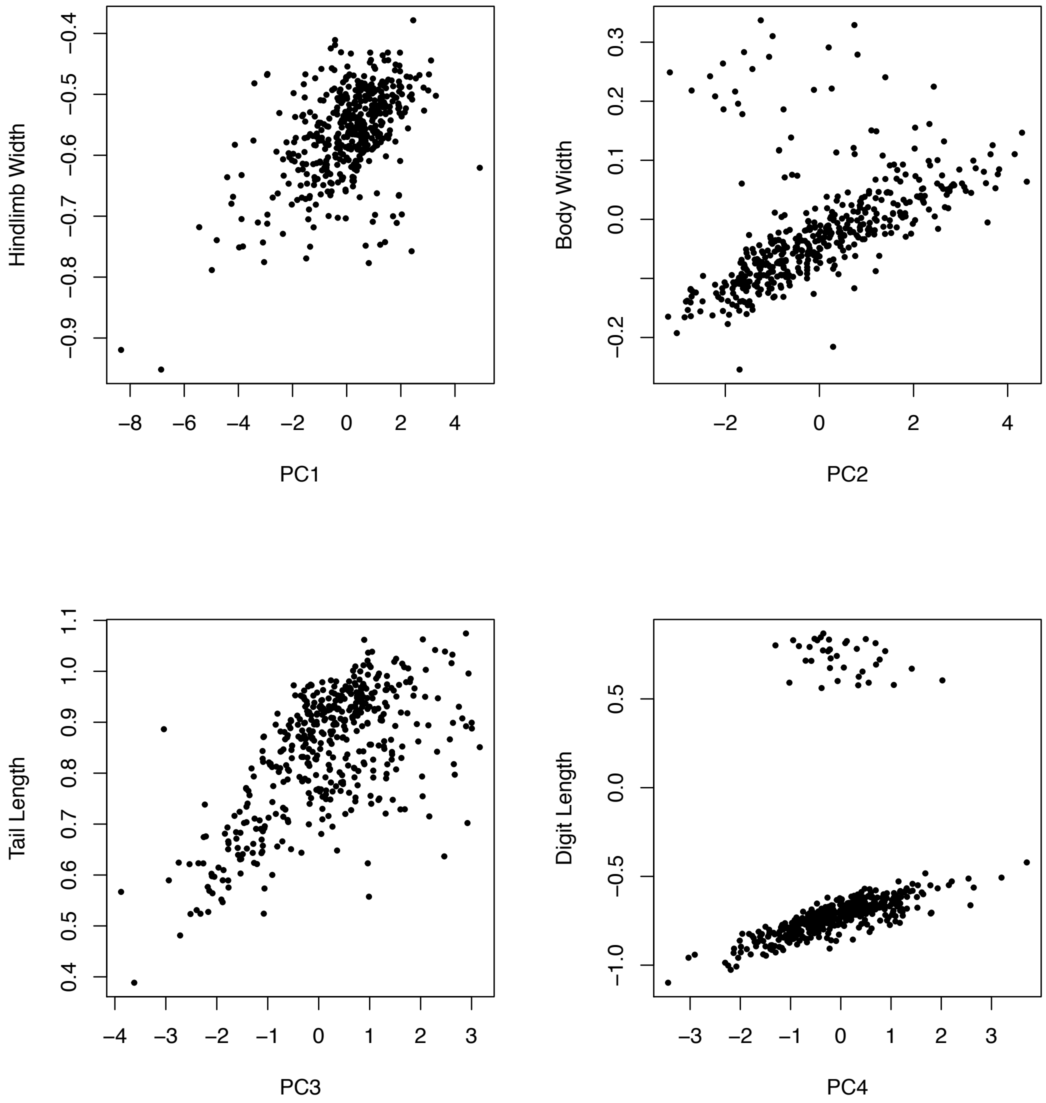

Supplement: S1 Fig — (PDF) [file pone.0216754.s001.pdf]
