# Supplementary material for: Ecological contributions to body shape evolution in salamanders of the genus Eurycea (Plethodontidae)
Source: PLoS One. 2019 May 15;14(5):e0216754. doi: 10.1371/journal.pone.0216754 (PMC6519905; doi:10.1371/journal.pone.0216754)
Supplement: S1 Table — (DOCX) [file pone.0216754.s002.docx]

Supplementary Table S1. Raw measurements from each sepecimen, together with identification, institution, and catalogue number.

|  |  |  |  | **Forelimb** | |  | **Hindlimb** | |  |  |  |
| --- | --- | --- | --- | --- | --- | --- | --- | --- | --- | --- | --- |
| **Institution** | **Catalog #** | **Species** | **Head Width** | **Length** | **Width** | **Body Width** | **Length** | **Width** | **Digit Length** | **Tail Length** | **Snout-Vent Length** |
| AMNH | 69032 | Eurycea aquatica | 6.596 | 7.664 | 1.343 | 6.368 | 9.659 | 2 | 0.857 | 42.676 | 36.807 |
| AMNH | 69033 | Eurycea aquatica | 6.525 | 8.656 | 1.302 | 6.521 | 9.682 | 1.802 | 0.46 | 44.696 | 41.854 |
| AMNH | 90687 | Eurycea aquatica | 5.454 | 6.337 | 1.37 | 4.639 | 9.036 | 1.435 | 0.648 | 36.587 | 36.99 |
| AMNH | 108781 | Eurycea aquatica | 6.659 | 7.704 | 1.21 | 6.024 | 8.715 | 1.837 | 1.205 | 41.699 | 42.159 |
| AMNH | 108782 | Eurycea aquatica | 5.803 | 7.809 | 1.201 | 5.589 | 9.619 | 1.761 | 0.999 | 30.493 | 40.378 |
| AMNH | 108783 | Eurycea aquatica | 6.017 | 8.232 | 1.186 | 6.385 | 9.325 | 1.865 | 0.99 | 36.24 | 40.081 |
| AMNH | 108784 | Eurycea aquatica | 5.556 | 4.624 | 0.937 | 5.587 | 5.293 | 0.917 | 0.724 | 25.907 | 32.118 |
| AMNH | 108785 | Eurycea aquatica | 4.846 | 6.908 | 1.013 | 4.809 | 8.244 | 1.463 | 0.663 | 22.73 | 33.252 |
| AMNH | 108786 | Eurycea aquatica | 6.361 | 6.786 | 1.069 | 5.828 | 6.739 | 1.503 | 0.846 | 25.766 | 32.603 |
| AMNH | 108787 | Eurycea aquatica | 4.841 | 4.999 | 0.629 | 3.909 | 4.234 | 0.907 | 0.656 | 19.91 | 24.657 |
| AMNH | 108788 | Eurycea aquatica | 5.048 | 6.829 | 1.01 | 4.421 | 9.008 | 1.145 | 1.102 | 21.35 | 30.818 |
| AMNH | 6632 | Eurycea bislineata | 5.862 | 5.518 | 0.873 | 5.103 | 8.95 | 1.626 | 1.483 | 23.406 | 42.618 |
| AMNH | 7540 | Eurycea bislineata | 5.8 | 5.918 | 1.156 | 5.096 | 8.055 | 1.283 | 1.074 | 22.317 | 38.189 |
| AMNH | 7543 | Eurycea bislineata | 5.269 | 5.212 | 1.281 | 4.961 | 6.981 | 1.376 | 0.813 | 35.74 | 32.817 |
| AMNH | 13099 | Eurycea bislineata | 4.8 | 5.516 | 1.121 | 5.363 | 7.972 | 1.855 | 1.187 | 45.4 | 41.929 |
| AMNH | 13100 | Eurycea bislineata | 6.404 | 4.529 | 1.407 | 6.271 | 5.194 | 1.331 | 1.058 | 44.798 | 47.714 |
| AMNH | 15748 | Eurycea bislineata | 4.864 | 6.013 | 0.966 | 4.055 | 7.556 | 1.425 | 0.962 | 28.567 | 32.072 |
| AMNH | 32864 | Eurycea bislineata | 4.647 | 6.225 | 0.926 | 4.881 | 7.393 | 1.226 | 0.801 | 37.494 | 33.456 |
| AMNH | 51541 | Eurycea bislineata | 4.809 | 5.091 | 1.369 | 5.515 | 7.849 | 1.667 | 0.943 | 34.418 | 36.436 |
| AMNH | 51697 | Eurycea bislineata | 4.669 | 4.384 | 1.371 | 4.013 | 6.545 | 1.395 | 1.028 | 40.424 | 25.77 |
| AMNH | 60780 | Eurycea bislineata | 5.091 | 6.272 | 1.333 | 6.8 | 8.236 | 1.674 | 0.773 | 33.587 | 38.623 |
| AMNH | 60781 | Eurycea bislineata | 5.023 | 5.48 | 1.32 | 6.444 | 8.652 | 1.618 | 1.136 | 32.311 | 38.273 |
| AMNH | 116809 | Eurycea bislineata | 5.729 | 6.862 | 1.643 | 6.763 | 7.903 | 2.129 | 0.898 | 54.252 | 46.117 |
| AMNH | 116810 | Eurycea bislineata | 5.17 | 7.504 | 1.164 | 4.812 | 7.909 | 1.641 | 1.073 | 49.009 | 39.356 |
| AMNH | 116811 | Eurycea bislineata | 5.758 | 6.83 | 1.672 | 5.973 | 9.356 | 1.846 | 1.36 | 61.378 | 44.261 |
| AMNH | 116812 | Eurycea bislineata | 4.856 | 7.55 | 1.14 | 5.405 | 8.238 | 1.75 | 0.841 | 44.381 | 36.811 |
| AMNH | 116813 | Eurycea bislineata | 4.5 | 5.956 | 1.02 | 4.195 | 7.736 | 1.353 | 1.106 | 41.202 | 30.659 |
| AMNH | 164483 | Eurycea bislineata | 5.974 | 7.377 | 1.338 | 6.129 | 8.612 | 1.873 | 1.319 | 51.906 | 41.782 |
| AMNH | 164484 | Eurycea bislineata | 5.896 | 7.734 | 1.39 | 5.859 | 10.23 | 1.804 | 1.442 | 41.826 | 42.704 |
| AMNH | 44200 | Eurycea cirrigera | 6.152 | 7.166 | 1.918 | 7.337 | 11.241 | 2.389 | 1.498 | 60.864 | 41.973 |
| AMNH | 50866 | Eurycea cirrigera | 4.98 | 5.362 | 1.363 | 5.139 | 6.78 | 1.749 | 1.042 | 53.248 | 34.472 |
| AMNH | 50867 | Eurycea cirrigera | 5.502 | 7.267 | 1.21 | 6.571 | 8.297 | 1.667 | 0.737 | 38.319 | 38.065 |
| AMNH | 50869 | Eurycea cirrigera | 5.766 | 7.072 | 1.42 | 5.899 | 9.201 | 2.149 | 1.059 | 47.309 | 40.323 |
| AMNH | 60784 | Eurycea cirrigera | 5.09 | 5.826 | 1.048 | 4.899 | 6.684 | 1.771 | 0.526 | 42.854 | 32.216 |
| AMNH | 62827 | Eurycea cirrigera | 5.907 | 7.028 | 1.509 | 6.377 | 9.068 | 2.075 | 1.341 | 45.157 | 37.28 |
| AMNH | 100411 | Eurycea cirrigera | 6.146 | 7.207 | 1.412 | 5.751 | 8.256 | 2.148 | 1.102 | 32.231 | 36.042 |
| AMNH | 100412 | Eurycea cirrigera | 7.018 | 8.088 | 1.51 | 6.48 | 9.04 | 1.943 | 1.503 | 51.787 | 40.952 |
| AMNH | 100415 | Eurycea cirrigera | 5.614 | 7.959 | 1.034 | 5.067 | 9.726 | 1.306 | 0.981 | 42.946 | 36.996 |
| AMNH | 135499 | Eurycea cirrigera | 3.458 | 4.606 | 0.631 | 3.389 | 5.775 | 0.841 | 0.619 | 39.811 | 27.57 |
| AMNH | 135500 | Eurycea cirrigera | 3.477 | 4.579 | 0.756 | 3.243 | 5.953 | 0.994 | 0.631 | 26.641 | 25.499 |
| AMNH | 135501 | Eurycea cirrigera | 3.304 | 3.871 | 0.657 | 3.013 | 6.302 | 1.143 | 0.624 | 25.623 | 24.329 |
| AMNH | 143130 | Eurycea cirrigera | 5.224 | 5.798 | 1.147 | 4.924 | 7.457 | 1.728 | 1.158 | 50.784 | 33.427 |
| AMNH | 143131 | Eurycea cirrigera | 5.108 | 6.705 | 1.116 | 4.81 | 9.243 | 1.656 | 1.43 | 45.408 | 30.927 |
| AMNH | 143133 | Eurycea cirrigera | 4.02 | 4.122 | 0.656 | 3.649 | 4.57 | 1.029 | 0.582 | 42.314 | 25.825 |
| AMNH | 143134 | Eurycea cirrigera | 3.648 | 4.666 | 0.978 | 3.833 | 5.866 | 1.02 | 0.876 | 35.24 | 26.397 |
| AMNH | 143135 | Eurycea cirrigera | 5.302 | 6.008 | 1.277 | 4.293 | 7.114 | 1.377 | 1.028 | 24.618 | 30.77 |
| AMNH | 143136 | Eurycea cirrigera | 6.006 | 7.098 | 1.504 | 5.547 | 9.602 | 2.025 | 0.927 | 48.744 | 36.11 |
| AMNH | 182150 | Eurycea cirrigera | 5.292 | 5.457 | 1.306 | 6.614 | 8.003 | 2.029 | 1.179 | 51.56 | 38.85 |
| AMNH | 182151 | Eurycea cirrigera | 5.329 | 7.499 | 1.197 | 5.656 | 8.309 | 1.684 | 1.255 | 44.949 | 39.814 |
| AMNH | 182154 | Eurycea cirrigera | 4.941 | 6.313 | 1.15 | 4.707 | 8.222 | 1.531 | 0.943 | 38.861 | 33.243 |
| AMNH | 182155 | Eurycea cirrigera | 5.364 | 5.753 | 1.075 | 4.623 | 6.151 | 1.496 | 0.953 | 37.393 | 32.596 |
| AMNH | 182156 | Eurycea cirrigera | 5.967 | 8.115 | 1.145 | 6.175 | 8.897 | 1.822 | 1.358 | 50.585 | 36.248 |
| AMNH | 3090 | Eurycea guttolineata | 8.439 | 8.283 | 1.747 | 10.291 | 12.06 | 2.838 | 1.386 | 81.382 | 58.541 |
| AMNH | 3997 | Eurycea guttolineata | 8.136 | 12.874 | 2.239 | 8.484 | 13.201 | 2.905 | 2.312 | 85.061 | 50.057 |
| AMNH | 3998 | Eurycea guttolineata | 6.696 | 11.197 | 2.221 | 9.107 | 12.703 | 3.143 | 1.656 | 88.048 | 54.398 |
| AMNH | 3999 | Eurycea guttolineata | 8.637 | 12.55 | 2.255 | 9.023 | 15.072 | 3.129 | 2.645 | 86.514 | 59.065 |
| AMNH | 21195 | Eurycea guttolineata | 7.297 | 11.377 | 1.778 | 7.161 | 13.229 | 2.597 | 1.683 | 81.635 | 52.149 |
| AMNH | 127073 | Eurycea guttolineata | 8.329 | 11.91 | 2.613 | 9.513 | 13.81 | 3.583 | 1.607 | 93.552 | 52.956 |
| AMNH | 127075 | Eurycea guttolineata | 9.094 | 11.847 | 2.147 | 10.526 | 14.692 | 4.21 | 1.285 | 92.233 | 59.267 |
| AMNH | 127076 | Eurycea guttolineata | 9.283 | 11.814 | 2.316 | 9.965 | 14.93 | 2.991 | 2.102 | 67.39 | 58.518 |
| AMNH | 127087 | Eurycea guttolineata | 7.21 | 10.188 | 1.648 | 6.442 | 11.553 | 2.363 | 1.252 | 52.617 | 45.636 |
| AMNH | 127088 | Eurycea guttolineata | 8.519 | 11.398 | 2.199 | 9.631 | 7.951 | 2.83 | 1.465 | 103.749 | 54.979 |
| AMNH | 127089 | Eurycea guttolineata | 7.837 | 11.338 | 2.012 | 9.484 | 12.812 | 2.943 | 1.556 | 102.123 | 55.773 |
| AMNH | 127092 | Eurycea guttolineata | 8.259 | 11.553 | 1.957 | 9.261 | 12.478 | 3.06 | 2.084 | 99.577 | 54.318 |
| AMNH | 127093 | Eurycea guttolineata | 8.874 | 11.895 | 2.292 | 9.4 | 11.902 | 3.154 | 1.356 | 87.293 | 53.873 |
| AMNH | 127102 | Eurycea guttolineata | 10.264 | 11.264 | 2.452 | 9.774 | 15.086 | 3.625 | 2.021 | 71.686 | 58.569 |
| AMNH | 182160 | Eurycea guttolineata | 6.978 | 9.025 | 1.738 | 5.926 | 12.176 | 2.002 | 1.55 | 55.687 | 41.405 |
| AMNH | 182161 | Eurycea guttolineata | 6.363 | 7.386 | 1.39 | 5.892 | 9.847 | 1.709 | 1.453 | 55.099 | 38.238 |
| AMNH | 187780 | Eurycea guttolineata | 7.818 | 9.88 | 2.006 | 9.165 | 15.731 | 2.845 | 2.616 | 80.138 | 54.632 |
| AMNH | 187781 | Eurycea guttolineata | 6.873 | 8.28 | 1.754 | 7.448 | 10.079 | 2.207 | 1.466 | 34.516 | 39.863 |
| AMNH | 187782 | Eurycea guttolineata | 7.32 | 12.39 | 1.66 | 7.126 | 13.978 | 1.979 | 1.741 | 56.304 | 46.076 |
| AMNH | 187783 | Eurycea guttolineata | 8.767 | 13.635 | 2.275 | 9.529 | 15.032 | 3.151 | 2.192 | 94.428 | 55.381 |
| AMNH | 187784 | Eurycea guttolineata | 8 | 12.136 | 1.836 | 8.485 | 13.779 | 3.113 | 1.626 | 51.937 | 53.589 |
| AMNH | 171578 | Eurycea junaluska | 4.141 | 5.944 | 0.778 | 3.748 | 7.576 | 1.357 | 0.646 | 43.76 | 30.914 |
| AMNH | 172184 | Eurycea junaluska | 5.801 | 9.147 | 0.997 | 6.429 | 9.713 | 1.605 | 1.168 | 34.19 | 40.11 |
| AMNH | 38057 | Eurycea l. longicauda | 6.859 | 11.758 | 1.84 | 5.537 | 14.402 | 1.981 | 1.203 | 87.513 | 54.983 |
| AMNH | 43662 | Eurycea l. longicauda | 6.109 | 7.662 | 1.428 | 5.414 | 11.561 | 1.885 | 1.747 | 73.492 | 41.951 |
| AMNH | 51698 | Eurycea l. longicauda | 7.633 | 13.903 | 2.212 | 10.087 | 15.2 | 3.356 | 1.607 | 93.134 | 51.571 |
| AMNH | 58232 | Eurycea l. longicauda | 9.138 | 13.048 | 2.145 | 8.461 | 15.117 | 2.581 | 1.796 | 87.131 | 54.254 |
| AMNH | 59810 | Eurycea l. longicauda | 7.391 | 10.025 | 1.433 | 5.951 | 14.01 | 2.387 | 0.794 | 61.405 | 51.295 |
| AMNH | 79728 | Eurycea l. longicauda | 6.285 | 10.997 | 1.278 | 5.652 | 11.723 | 1.991 | 0.741 | 24.404 | 37.336 |
| AMNH | 89783 | Eurycea l. longicauda | 8.072 | 13.612 | 1.769 | 8.154 | 16.433 | 2.409 | 1.919 | 101.849 | 57.656 |
| AMNH | 99284 | Eurycea l. longicauda | 7.746 | 11.152 | 2.268 | 8.14 | 15.684 | 2.784 | 1.715 | 97.844 | 57.65 |
| AMNH | 99286 | Eurycea l. longicauda | 8.415 | 14.088 | 1.76 | 6.858 | 18.345 | 2.386 | 1.458 | 59.503 | 62.019 |
| AMNH | 112043 | Eurycea l. longicauda | 5.893 | 9.206 | 1.553 | 4.935 | 12.257 | 1.741 | 1.015 | 61.414 | 41.73 |
| AMNH | 112044 | Eurycea l. longicauda | 5.75 | 9.066 | 1.25 | 5.344 | 9.953 | 2.057 | 1.041 | 61.321 | 40.555 |
| AMNH | 112045 | Eurycea l. longicauda | 5.809 | 10.127 | 1.173 | 5.005 | 11.166 | 1.921 | 1.38 | 69.868 | 49.388 |
| AMNH | 114012 | Eurycea l. longicauda | 6.994 | 9.902 | 1.986 | 8.222 | 12.442 | 2.682 | 1.496 | 88.872 | 54.015 |
| AMNH | 114385 | Eurycea l. longicauda | 7.26 | 15.338 | 1.992 | 9.043 | 13.504 | 2.411 | 1.207 | 77.347 | 53.23 |
| AMNH | 114386 | Eurycea l. longicauda | 5.82 | 10.936 | 1.397 | 5.748 | 12.109 | 2.419 | 1.041 | 61.822 | 39.017 |
| AMNH | 115838 | Eurycea l. longicauda | 7.181 | 12.544 | 1.692 | 6.415 | 13.788 | 1.916 | 1.817 | 80.307 | 49.503 |
| AMNH | 135240 | Eurycea l. longicauda | 7.924 | 14.599 | 1.816 | 8.067 | 15.588 | 2.837 | 1.598 | 87.949 | 58.252 |
| AMNH | 135241 | Eurycea l. longicauda | 7.423 | 12.282 | 1.923 | 8.129 | 14.971 | 2.493 | 1.614 | 79.745 | 51.717 |
| AMNH | 135782 | Eurycea l. longicauda | 6.495 | 12.608 | 1.861 | 6.806 | 15.264 | 2.426 | 2.079 | 93.709 | 48.254 |
| AMNH | 136646 | Eurycea l. longicauda | 7.23 | 11.555 | 1.708 | 6.453 | 13.847 | 2.37 | 2.007 | 60.713 | 50.936 |
| AMNH | 148944 | Eurycea l. longicauda | 6.659 | 9.453 | 1.481 | 5.831 | 12.119 | 1.719 | 1.447 | 55.706 | 42.587 |
| AMNH | 148950 | Eurycea l. longicauda | 6.399 | 10.474 | 1.485 | 6.365 | 12.847 | 1.838 | 1.089 | 64.379 | 45.55 |
| AMNH | 151305 | Eurycea l. longicauda | 7.889 | 12.968 | 1.826 | 7.231 | 13.499 | 2.235 | 1.737 | 74.055 | 54.626 |
| AMNH | 40345 | Eurycea l. melanopleura | 7.62 | 10.747 | 1.718 | 6.213 | 13.113 | 2.111 | 1.937 | 74.188 | 49.066 |
| AMNH | 40346 | Eurycea l. melanopleura | 8.626 | 11.891 | 1.638 | 6.858 | 11.772 | 2.796 | 1.577 | 80.155 | 44.461 |
| AMNH | 40347 | Eurycea l. melanopleura | 7.692 | 9.978 | 1.522 | 6.396 | 12.388 | 2.021 | 1.102 | 64.487 | 47.485 |
| AMNH | 40349 | Eurycea l. melanopleura | 6.261 | 10.283 | 1.236 | 6.171 | 10.886 | 1.757 | 1.708 | 36.223 | 40.633 |
| AMNH | 40351 | Eurycea l. melanopleura | 7.402 | 10.454 | 1.919 | 7.08 | 12.816 | 2.195 | 1.388 | 82.262 | 49.768 |
| AMNH | 40354 | Eurycea l. melanopleura | 6.273 | 8.259 | 1.519 | 5.715 | 11.478 | 1.664 | 1.1 | 62.701 | 55.342 |
| AMNH | 40356 | Eurycea l. melanopleura | 8.712 | 9.951 | 1.524 | 7.28 | 12.056 | 2.576 | 1.685 | 83.172 | 45.669 |
| AMNH | 52071 | Eurycea l. melanopleura | 9.054 | 8.712 | 2.087 | 8.768 | 12.798 | 2.697 | 1.959 | 83.032 | 54.316 |
| AMNH | 52072 | Eurycea l. melanopleura | 8.617 | 12.106 | 2.318 | 9.463 | 13.303 | 3.309 | 1.55 | 53.551 | 56.266 |
| AMNH | 52073 | Eurycea l. melanopleura | 8.293 | 11.984 | 2.082 | 10.27 | 15.429 | 2.973 | 1.802 | 86.971 | 57.069 |
| AMNH | 52075 | Eurycea l. melanopleura | 7.898 | 9.522 | 1.524 | 7.377 | 12.393 | 2.441 | 1.175 | 60.643 | 43.999 |
| AMNH | 52077 | Eurycea l. melanopleura | 9.649 | 12.335 | 2.349 | 10.192 | 13.62 | 3.174 | 1.954 | 74.178 | 58.875 |
| AMNH | 52078 | Eurycea l. melanopleura | 8.505 | 12.655 | 1.903 | 9.481 | 14.589 | 2.927 | 1.554 | 67.296 | 60.688 |
| AMNH | 59797 | Eurycea l. melanopleura | 6.142 | 9.774 | 1.086 | 5.621 | 11.682 | 1.595 | 1.523 | 54.255 | 41.083 |
| AMNH | 59798 | Eurycea l. melanopleura | 5.64 | 7.349 | 1.182 | 4.93 | 10.199 | 1.595 | 0.8 | 40.804 | 36.57 |
| AMNH | 59799 | Eurycea l. melanopleura | 6.239 | 8.882 | 1.307 | 4.872 | 11.034 | 1.704 | 1.301 | 32.847 | 40.588 |
| AMNH | 59800 | Eurycea l. melanopleura | 5.588 | 8.096 | 1.03 | 4.83 | 9.752 | 1.321 | 0.895 | 41.353 | 35.52 |
| AMNH | 149001 | Eurycea l. melanopleura | 5.213 | 8.581 | 1.055 | 4.727 | 9.625 | 1.698 | 1.069 | 44.608 | 35.411 |
| AMNH | 149201 | Eurycea l. melanopleura | 7.794 | 11.172 | 1.656 | 7.241 | 12.367 | 2.551 | 1.262 | 61.83 | 45.241 |
| AMNH | 149212 | Eurycea l. melanopleura | 8.786 | 11.507 | 1.956 | 10.484 | 13.034 | 2.795 | 1.684 | 87.046 | 53.768 |
| AMNH | 149230 | Eurycea l. melanopleura | 7.97 | 12.033 | 2.284 | 9.056 | 13.588 | 3.044 | 1.566 | 77.246 | 56.029 |
| AMNH | 149249 | Eurycea l. melanopleura | 7.532 | 11.992 | 2.02 | 7.827 | 12.347 | 2.511 | 1.197 | 73.182 | 48.489 |
| NMNH | 123594 | Eurycea latitans | 7.991 | 6.943 | 1.546 | 7.115 | 8.871 | 1.895 | 1.477 | 45.758 | 46.945 |
| NMNH | 545379 | Eurycea latitans | 8.499 | 8.677 | 1.401 | 7.199 | 8.519 | 1.227 | 1.779 | 41.844 | 41.392 |
| AMNH | 11867 | Eurycea lucifuga | 8.835 | 13.762 | 2.078 | 7.478 | 17.943 | 2.627 | 1.742 | 72.338 | 53.867 |
| AMNH | 11868 | Eurycea lucifuga | 9.114 | 14.758 | 2.387 | 8.402 | 20.227 | 2.47 | 2.772 | 87.605 | 63.245 |
| AMNH | 16826 | Eurycea lucifuga | 10.045 | 17.462 | 2.189 | 8.95 | 19.346 | 2.529 | 2.191 | 78.219 | 64.906 |
| AMNH | 22915 | Eurycea lucifuga | 6.289 | 10.881 | 1.473 | 4.878 | 9.346 | 1.288 | 1.562 | 60.8 | 37.313 |
| AMNH | 22916 | Eurycea lucifuga | 8.262 | 16.264 | 2.227 | 9.052 | 16.463 | 2.993 | 1.963 | 80.656 | 55.416 |
| AMNH | 22917 | Eurycea lucifuga | 8.599 | 13.923 | 1.873 | 7.828 | 13.071 | 2.066 | 2.324 | 70.119 | 46.463 |
| AMNH | 22918 | Eurycea lucifuga | 5.751 | 8.802 | 1.125 | 5.171 | 10.6 | 2.305 | 1.44 | 22.33 | 39.554 |
| AMNH | 22920 | Eurycea lucifuga | 8.752 | 13.1 | 2.084 | 8.638 | 14.213 | 2.783 | 2.228 | 82.81 | 55.115 |
| AMNH | 22921 | Eurycea lucifuga | 6.841 | 12.186 | 1.75 | 5.777 | 11.454 | 2.104 | 1.884 | 45.598 | 62.327 |
| AMNH | 22922 | Eurycea lucifuga | 7.085 | 10.437 | 1.161 | 5.575 | 11.326 | 2.071 | 1.731 | 56.376 | 42.017 |
| AMNH | 32175 | Eurycea lucifuga | 6.137 | 9.108 | 1.222 | 4.474 | 10.765 | 1.796 | 1.199 | 44.7 | 35.17 |
| AMNH | 32176 | Eurycea lucifuga | 5.73 | 8.213 | 1.201 | 4.819 | 10.41 | 1.682 | 1.023 | 43.592 | 35.055 |
| AMNH | 33407 | Eurycea lucifuga | 9.573 | 15.588 | 2.152 | 7.873 | 15.226 | 2.797 | 1.741 | 83.79 | 60.548 |
| AMNH | 33408 | Eurycea lucifuga | 8.628 | 14.404 | 2.035 | 6.82 | 14.613 | 2.577 | 1.36 | 83.463 | 57.146 |
| AMNH | 33409 | Eurycea lucifuga | 8.642 | 14.66 | 2.128 | 7.829 | 16.127 | 2.889 | 2.182 | 83.135 | 56.52 |
| AMNH | 36112 | Eurycea lucifuga | 5.479 | 6.601 | 1.201 | 4.549 | 8.843 | 1.704 | 0.814 | 19.463 | 35.544 |
| AMNH | 36113 | Eurycea lucifuga | 5.666 | 7.943 | 1.208 | 4.785 | 9.452 | 1.899 | 1.255 | 42.509 | 34.784 |
| AMNH | 36115 | Eurycea lucifuga | 5.854 | 9.905 | 1.269 | 4.624 | 10.76 | 1.331 | 1.012 | 39.087 | 35.959 |
| AMNH | 36116 | Eurycea lucifuga | 5.895 | 8.599 | 1.193 | 5.216 | 10.132 | 1.673 | 1.039 | 17.884 | 36.321 |
| AMNH | 36120 | Eurycea lucifuga | 6.144 | 10.216 | 1.487 | 4.942 | 9.36 | 1.926 | 1.129 | 36.264 | 36.771 |
| AMNH | 38500 | Eurycea lucifuga | 10.297 | 17.352 | 2.507 | 8.234 | 19.074 | 2.435 | 2.231 | 63.567 | 62.796 |
| AMNH | 40342 | Eurycea lucifuga | 9.337 | 16.878 | 2.469 | 7.99 | 18.258 | 2.556 | 1.618 | 64.874 | 60.08 |
| AMNH | 41467 | Eurycea lucifuga | 6.947 | 12.175 | 1.817 | 6.946 | 12.443 | 2.964 | 1.951 | 70.418 | 47.096 |
| AMNH | 41786 | Eurycea lucifuga | 8.038 | 13.998 | 1.702 | 6.476 | 16.564 | 2.31 | 2.816 | 37.623 | 49.144 |
| AMNH | 50036 | Eurycea lucifuga | 6.985 | 12.247 | 1.569 | 6.095 | 13.82 | 2.147 | 1.319 | 62.894 | 44.728 |
| AMNH | 52068 | Eurycea lucifuga | 10.278 | 16.933 | 3.042 | 10.61 | 15.891 | 3.282 | 1.658 | 87.868 | 64.13 |
| AMNH | 52069 | Eurycea lucifuga | 8.639 | 14.282 | 1.736 | 7.631 | 14.167 | 2.619 | 1.814 | 72.62 | 51.597 |
| AMNH | 52104 | Eurycea lucifuga | 10.491 | 16.401 | 2.258 | 10.586 | 17.421 | 3.174 | 1.99 | 86.751 | 61.561 |
| AMNH | 52105 | Eurycea lucifuga | 9.854 | 15.691 | 2.75 | 10.368 | 15.457 | 3.523 | 1.805 | 80.444 | 57.544 |
| AMNH | 52106 | Eurycea lucifuga | 10.251 | 15.926 | 2.153 | 7.403 | 18.124 | 3.525 | 2.005 | 68.362 | 61.454 |
| AMNH | 52107 | Eurycea lucifuga | 10.173 | 15.134 | 2.688 | 7.26 | 19.516 | 2.968 | 2.141 | 90.776 | 65.173 |
| AMNH | 52108 | Eurycea lucifuga | 9.806 | 15.095 | 1.865 | 8.91 | 15.143 | 3.194 | 1.444 | 89.484 | 63.845 |
| AMNH | 52109 | Eurycea lucifuga | 9.674 | 15.36 | 2.13 | 9.039 | 18.062 | 2.765 | 1.543 | 86.221 | 59.878 |
| AMNH | 52110 | Eurycea lucifuga | 10.704 | 13.747 | 2.104 | 9.621 | 15.946 | 3.178 | 1.88 | 98.029 | 63.134 |
| AMNH | 52111 | Eurycea lucifuga | 10.397 | 12.453 | 1.953 | 7.084 | 14.422 | 3.004 | 1.71 | 63.746 | 54.043 |
| AMNH | 52112 | Eurycea lucifuga | 8.406 | 12.906 | 1.959 | 7.27 | 13.592 | 2.628 | 1.836 | 76.394 | 51.894 |
| AMNH | 52113 | Eurycea lucifuga | 9.035 | 14.87 | 1.901 | 7.787 | 14.286 | 2.353 | 2.083 | 75.554 | 57.213 |
| AMNH | 52114 | Eurycea lucifuga | 7.847 | 12.663 | 1.513 | 6.243 | 15.845 | 2.734 | 2.395 | 69.316 | 51.308 |
| AMNH | 52115 | Eurycea lucifuga | 8.653 | 14.162 | 1.919 | 6.493 | 13.455 | 2.762 | 1.574 | 87.37 | 54.992 |
| AMNH | 52117 | Eurycea lucifuga | 9.951 | 17.052 | 2.243 | 8.677 | 17.232 | 2.711 | 1.826 | 65.411 | 57.671 |
| AMNH | 52118 | Eurycea lucifuga | 9.046 | 12.949 | 1.926 | 8.073 | 13.624 | 1.786 | 2.118 | 75.353 | 55.074 |
| AMNH | 52119 | Eurycea lucifuga | 9.602 | 16.117 | 2.283 | 8.855 | 16.591 | 2.938 | 1.678 | 89.114 | 62.928 |
| AMNH | 52121 | Eurycea lucifuga | 9.743 | 16.259 | 2.429 | 8.228 | 19.049 | 3.449 | 1.987 | 65.683 | 60.547 |
| AMNH | 52443 | Eurycea lucifuga | 10.743 | 17.62 | 2.661 | 8.708 | 18.105 | 3.555 | 1.957 | 73.129 | 72.792 |
| AMNH | 52444 | Eurycea lucifuga | 10.357 | 13.532 | 2.728 | 7.985 | 14.888 | 2.745 | 1.838 | 75.317 | 60.682 |
| AMNH | 52445 | Eurycea lucifuga | 10.248 | 13.398 | 2.194 | 10.52 | 16.237 | 3.181 | 1.348 | 49.48 | 58.432 |
| AMNH | 52446 | Eurycea lucifuga | 11.948 | 16.155 | 3.284 | 11.944 | 15.76 | 4.098 | 2.489 | 95.947 | 68.362 |
| AMNH | 52475 | Eurycea lucifuga | 9.555 | 12.817 | 2.156 | 8.004 | 15.557 | 3.29 | 1.684 | 90.28 | 57.608 |
| AMNH | 52527 | Eurycea lucifuga | 9.038 | 13.866 | 1.661 | 5.681 | 15.204 | 1.903 | 1.991 | 61.356 | 44.06 |
| AMNH | 54554 | Eurycea lucifuga | 11.179 | 14.243 | 2.55 | 7.484 | 17.492 | 3.168 | 1.493 | 58.82 | 68.111 |
| AMNH | 54555 | Eurycea lucifuga | 9.748 | 15.288 | 2.157 | 7.053 | 15.356 | 2.411 | 1.561 | 81.683 | 62.531 |
| AMNH | 56367 | Eurycea lucifuga | 7.081 | 11.155 | 1.206 | 5.408 | 13.487 | 1.936 | 1.668 | 52.959 | 44.481 |
| AMNH | 56368 | Eurycea lucifuga | 9.97 | 18.553 | 2.136 | 8.033 | 19.41 | 2.738 | 1.854 | 65.803 | 64.546 |
| AMNH | 58233 | Eurycea lucifuga | 9.843 | 16.616 | 1.92 | 9.065 | 21.288 | 3.119 | 2.327 | 111.287 | 61.433 |
| AMNH | 58234 | Eurycea lucifuga | 9.627 | 14.66 | 2.246 | 7.74 | 17.59 | 2.924 | 2.926 | 112.952 | 60.326 |
| AMNH | 58235 | Eurycea lucifuga | 9.706 | 16.969 | 1.763 | 7.695 | 17.375 | 2.949 | 2.652 | 97.394 | 57.321 |
| AMNH | 58236 | Eurycea lucifuga | 7.199 | 14.036 | 2.215 | 7.212 | 15.273 | 2.873 | 2.362 | 83.224 | 49.416 |
| AMNH | 59020 | Eurycea lucifuga | 9.949 | 16.082 | 2.142 | 7.899 | 14.404 | 2.294 | 2.254 | 100.55 | 62.842 |
| AMNH | 59021 | Eurycea lucifuga | 9.577 | 11.977 | 2.144 | 8.923 | 13.735 | 2.777 | 2.285 | 95.046 | 58.132 |
| AMNH | 59022 | Eurycea lucifuga | 8.323 | 12.185 | 2.028 | 6.822 | 16.135 | 2.6 | 2.096 | 83.843 | 54.214 |
| AMNH | 59758 | Eurycea lucifuga | 11.37 | 16.852 | 2.448 | 8.768 | 16.981 | 3.076 | 1.445 | 87.448 | 66.974 |
| AMNH | 59759 | Eurycea lucifuga | 9.307 | 15.717 | 1.663 | 7.019 | 18.186 | 2.619 | 2.306 | 62.508 | 52.743 |
| AMNH | 59760 | Eurycea lucifuga | 9.685 | 15.949 | 2.489 | 7.204 | 16.144 | 2.464 | 1.948 | 45.587 | 60.544 |
| AMNH | 59761 | Eurycea lucifuga | 8.92 | 15.7 | 2.109 | 7.063 | 18.241 | 2.487 | 2.673 | 89.711 | 59.922 |
| AMNH | 59762 | Eurycea lucifuga | 7.506 | 11.725 | 1.669 | 5.282 | 14.393 | 1.861 | 1.598 | 61.957 | 45.279 |
| AMNH | 59764 | Eurycea lucifuga | 11.543 | 16.953 | 2.635 | 9.708 | 18.043 | 3.287 | 3.113 | 79.978 | 62.149 |
| AMNH | 59765 | Eurycea lucifuga | 8.696 | 11.597 | 1.93 | 7.039 | 13.93 | 2.152 | 1.76 | 74.496 | 55.809 |
| AMNH | 59767 | Eurycea lucifuga | 11.14 | 17.152 | 2.499 | 9.193 | 16.625 | 3.04 | 1.27 | 76.619 | 61.347 |
| AMNH | 59768 | Eurycea lucifuga | 8.309 | 14.032 | 1.721 | 6.803 | 15.873 | 2.292 | 1.933 | 77.071 | 54.986 |
| AMNH | 59769 | Eurycea lucifuga | 8.67 | 14.313 | 1.984 | 8.171 | 16.535 | 2.687 | 1.584 | 90.558 | 58.845 |
| AMNH | 59770 | Eurycea lucifuga | 10.702 | 15.783 | 2.314 | 7.951 | 16.923 | 2.65 | 2.598 | 100.495 | 71.033 |
| AMNH | 59771 | Eurycea lucifuga | 10.17 | 15.257 | 2.206 | 7.253 | 16.894 | 2.845 | 2.543 | 76.438 | 59.669 |
| AMNH | 59772 | Eurycea lucifuga | 8.507 | 13.026 | 1.813 | 6.025 | 13.548 | 2.352 | 1.934 | 65.271 | 53.001 |
| AMNH | 59780 | Eurycea lucifuga | 11.349 | 13.46 | 2.057 | 9.513 | 18.492 | 2.911 | 2.17 | 90.315 | 58.543 |
| AMNH | 59781 | Eurycea lucifuga | 12.442 | 17.857 | 3.02 | 12.245 | 17.289 | 4.107 | 2.615 | 84.226 | 66.225 |
| AMNH | 59782 | Eurycea lucifuga | 10.478 | 14.301 | 2.661 | 10.406 | 17.016 | 2.903 | 1.666 | 68.78 | 59.79 |
| AMNH | 59803 | Eurycea lucifuga | 8.874 | 13.955 | 8.144 | 6.166 | 9.843 | 2.428 | 1.754 | 78.01 | 55.488 |
| AMNH | 59804 | Eurycea lucifuga | 9.575 | 17.61 | 2.076 | 8.774 | 18.407 | 3.063 | 1.84 | 70.975 | 65.827 |
| AMNH | 59805 | Eurycea lucifuga | 11.045 | 17.125 | 2.399 | 8.515 | 16.043 | 3.219 | 2.196 | 81.658 | 64.328 |
| AMNH | 59806 | Eurycea lucifuga | 10.402 | 14.001 | 2.382 | 8.31 | 17.322 | 3.372 | 1.701 | 81.556 | 61.264 |
| AMNH | 59807 | Eurycea lucifuga | 9.513 | 15.933 | 2.39 | 8.618 | 18.279 | 3.248 | 1.904 | 91.709 | 59.594 |
| AMNH | 137469 | Eurycea lucifuga | 9.785 | 18.695 | 2.085 | 9.018 | 17.12 | 2.706 | 2.373 | 77.406 | 65.295 |
| AMNH | 137470 | Eurycea lucifuga | 10.119 | 15.641 | 2.003 | 8.227 | 20.534 | 2.357 | 2.336 | 98.909 | 66.436 |
| AMNH | 137471 | Eurycea lucifuga | 9.758 | 18.251 | 1.487 | 7.804 | 19.245 | 2.498 | 2.446 | 82.319 | 63.379 |
| AMNH | 137472 | Eurycea lucifuga | 10.462 | 17.448 | 2.443 | 8.883 | 20.597 | 2.574 | 2.546 | 89.321 | 65.703 |
| AMNH | 137473 | Eurycea lucifuga | 9.583 | 16.614 | 1.423 | 6.607 | 16.783 | 2.25 | 2.695 | 46.575 | 60.052 |
| AMNH | 143530 | Eurycea lucifuga | 8.386 | 12.359 | 2.001 | 7.814 | 14.892 | 2.94 | 2.093 | 74.072 | 53.145 |
| AMNH | 143532 | Eurycea lucifuga | 10.463 | 15.148 | 2.152 | 9.828 | 16.696 | 2.84 | 2.453 | 44.834 | 64.299 |
| AMNH | 143533 | Eurycea lucifuga | 8.025 | 12.146 | 1.495 | 6.425 | 15.303 | 2.094 | 1.56 | 82.282 | 54.298 |
| AMNH | 143534 | Eurycea lucifuga | 11.342 | 15.318 | 2.426 | 8.268 | 18.668 | 4.103 | 2.118 | 71.429 | 62.393 |
| AMNH | 143535 | Eurycea lucifuga | 7.701 | 12.23 | 1.552 | 6.355 | 15.195 | 2.798 | 1.643 | 53.655 | 48.355 |
| AMNH | 143536 | Eurycea lucifuga | 8.836 | 13.615 | 1.588 | 6.5 | 15.323 | 2.572 | 1.925 | 75.323 | 58.023 |
| AMNH | 143537 | Eurycea lucifuga | 9.026 | 14.207 | 1.953 | 7.592 | 15.52 | 2.657 | 2.054 | 58.387 | 50.401 |
| AMNH | 143539 | Eurycea lucifuga | 9.979 | 16.261 | 2.514 | 7.97 | 16.788 | 3.136 | 2.021 | 58.636 | 62.492 |
| AMNH | 143540 | Eurycea lucifuga | 10.03 | 15.801 | 2.238 | 7.711 | 17.335 | 2.858 | 2.418 | 83.866 | 58.229 |
| AMNH | 143541 | Eurycea lucifuga | 10.909 | 17.206 | 2.814 | 8.283 | 18.966 | 3.75 | 1.32 | 36.301 | 58.08 |
| AMNH | 143542 | Eurycea lucifuga | 10.483 | 13.325 | 2.025 | 7.933 | 14.178 | 3.251 | 2.056 | 74.078 | 57.939 |
| AMNH | 143543 | Eurycea lucifuga | 9.08 | 14.55 | 2.138 | 8.493 | 17.079 | 2.902 | 1.924 | 47.94 | 56.601 |
| AMNH | 143545 | Eurycea lucifuga | 10.735 | 12.88 | 2.309 | 8.474 | 17.499 | 3.09 | 2.045 | 65.707 | 63.167 |
| AMNH | 143547 | Eurycea lucifuga | 9.182 | 11.639 | 1.805 | 8.366 | 13.759 | 2.414 | 2.338 | 82.181 | 59.8 |
| AMNH | 143548 | Eurycea lucifuga | 10.002 | 16.141 | 2.095 | 8.379 | 17.307 | 3.759 | 1.434 | 93.631 | 59.452 |
| AMNH | 143549 | Eurycea lucifuga | 9.882 | 14.817 | 2.166 | 7.482 | 14.943 | 3.223 | 2.063 | 44.798 | 62.504 |
| AMNH | 143552 | Eurycea lucifuga | 8.214 | 12.744 | 1.876 | 8.514 | 13.741 | 2.63 | 2.515 | 81.115 | 57.078 |
| AMNH | 143553 | Eurycea lucifuga | 6.574 | 14.214 | 1.95 | 7.743 | 13.677 | 1.891 | 1.902 | 81.083 | 57.566 |
| AMNH | 143554 | Eurycea lucifuga | 8.041 | 12.743 | 1.787 | 6.697 | 17.589 | 2.362 | 2.464 | 73.88 | 50.818 |
| AMNH | 143555 | Eurycea lucifuga | 7.85 | 12.4 | 1.957 | 6.718 | 14.624 | 2.399 | 1.626 | 59.124 | 50.298 |
| AMNH | 143556 | Eurycea lucifuga | 6.589 | 9.574 | 0.978 | 5.36 | 13.938 | 2.043 | 1.609 | 71.062 | 49.159 |
| AMNH | 143557 | Eurycea lucifuga | 8.955 | 13.147 | 2.549 | 7.755 | 18.593 | 2.973 | 1.561 | 50.697 | 49.244 |
| AMNH | 143558 | Eurycea lucifuga | 10.425 | 12.598 | 1.904 | 8.034 | 11.013 | 3.448 | 1.896 | 89.405 | 61.082 |
| AMNH | 143559 | Eurycea lucifuga | 7.832 | 14.936 | 1.333 | 6.034 | 17.038 | 1.885 | 2.117 | 88.793 | 56.807 |
| AMNH | 143560 | Eurycea lucifuga | 9.462 | 16.111 | 1.825 | 7.624 | 16.25 | 3.368 | 2.241 | 86.722 | 58.721 |
| AMNH | 143561 | Eurycea lucifuga | 10.039 | 15.521 | 2.329 | 8.375 | 15.871 | 3.504 | 2.373 | 78.51 | 57.3 |
| AMNH | 143562 | Eurycea lucifuga | 8.167 | 11.716 | 1.83 | 6.747 | 14.169 | 2.735 | 1.191 | 73.148 | 51.369 |
| AMNH | 143563 | Eurycea lucifuga | 10.271 | 13.971 | 1.986 | 8.794 | 15.583 | 3.216 | 2.04 | 88.6 | 59.777 |
| AMNH | 143564 | Eurycea lucifuga | 7.97 | 15.332 | 1.735 | 5.886 | 15.421 | 3.107 | 2.039 | 72.532 | 51.914 |
| AMNH | 143565 | Eurycea lucifuga | 9.258 | 12.581 | 1.694 | 7.139 | 13.251 | 2.812 | 1.457 | 73.747 | 56.157 |
| AMNH | 143566 | Eurycea lucifuga | 7.393 | 13.879 | 2.062 | 6.73 | 15.838 | 3.111 | 2.187 | 70.714 | 48.198 |
| AMNH | 143567 | Eurycea lucifuga | 8.658 | 13.403 | 1.834 | 4.439 | 9.598 | 2.866 | 1.57 | 65.834 | 48.489 |
| AMNH | 143568 | Eurycea lucifuga | 8.85 | 12.962 | 2.313 | 7.242 | 15.296 | 2.711 | 1.591 | 74.059 | 54.036 |
| AMNH | 143569 | Eurycea lucifuga | 7.312 | 10.6 | 1.839 | 6.537 | 15.152 | 2.725 | 1.675 | 67.174 | 45.214 |
| AMNH | 143570 | Eurycea lucifuga | 9.894 | 16.72 | 2.323 | 8.365 | 18.454 | 2.87 | 1.508 | 69.779 | 56.122 |
| AMNH | 143573 | Eurycea lucifuga | 9.644 | 12.236 | 2.447 | 7.002 | 15.183 | 2.576 | 1.619 | 89.082 | 53.277 |
| AMNH | 143574 | Eurycea lucifuga | 9.434 | 13.015 | 1.808 | 7.142 | 16.121 | 2.452 | 1.622 | 79.805 | 53.557 |
| AMNH | 143575 | Eurycea lucifuga | 8.096 | 11.406 | 1.877 | 5.959 | 15.389 | 2.308 | 1.598 | 58.546 | 48.468 |
| AMNH | 143576 | Eurycea lucifuga | 8.779 | 13.452 | 1.841 | 6.018 | 14.972 | 2.553 | 1.597 | 72.889 | 50.533 |
| AMNH | 143577 | Eurycea lucifuga | 9.033 | 13.316 | 2.07 | 7.64 | 17.159 | 2.796 | 1.298 | 60.537 | 53.441 |
| AMNH | 143578 | Eurycea lucifuga | 10.283 | 15.83 | 2.37 | 8.68 | 16.283 | 3.241 | 1.82 | 77.822 | 61.63 |
| AMNH | 143579 | Eurycea lucifuga | 10.546 | 16.943 | 2.637 | 9.462 | 18.707 | 3.033 | 1.82 | 99.526 | 59.269 |
| AMNH | 143580 | Eurycea lucifuga | 9.653 | 14.189 | 2.398 | 7.195 | 16.168 | 3.049 | 1.974 | 87.148 | 61.679 |
| AMNH | 143582 | Eurycea lucifuga | 7.807 | 12.654 | 1.768 | 6.244 | 13.902 | 2.221 | 1.583 | 51.365 | 46.775 |
| AMNH | 143583 | Eurycea lucifuga | 9.582 | 16.395 | 2.178 | 7.391 | 15.697 | 2.884 | 2.249 | 21.468 | 55.945 |
| AMNH | 143584 | Eurycea lucifuga | 8.378 | 15.014 | 1.727 | 7.195 | 14.738 | 2.334 | 1.837 | 72.467 | 50.98 |
| AMNH | 143585 | Eurycea lucifuga | 7.745 | 12.745 | 1.65 | 5.28 | 14.466 | 1.788 | 1.626 | 32.056 | 49.879 |
| AMNH | 143588 | Eurycea lucifuga | 6.928 | 11.422 | 1.187 | 5.359 | 11.242 | 1.835 | 1.355 | 26.582 | 43.049 |
| AMNH | 143589 | Eurycea lucifuga | 10.32 | 16.165 | 2.447 | 7.715 | 18.073 | 3.563 | 1.948 | 47.928 | 60.009 |
| AMNH | 155760 | Eurycea lucifuga | 10.837 | 14.761 | 2.78 | 8.583 | 18.214 | 3.438 | 1.775 | 100.342 | 65.176 |
| AMNH | 155761 | Eurycea lucifuga | 9.833 | 16.186 | 2.138 | 9.522 | 17.33 | 2.72 | 1.864 | 88.877 | 60.004 |
| AMNH | 182172 | Eurycea lucifuga | 8.97 | 15.588 | 2.196 | 8.599 | 16.512 | 2.84 | 2.283 | 75.127 | 55.547 |
| AMNH | 182173 | Eurycea lucifuga | 7.836 | 9.328 | 1.842 | 8.731 | 15.839 | 2.919 | 2.743 | 80.571 | 55.312 |
| AMNH | 187787 | Eurycea lucifuga | 8.655 | 12.518 | 1.617 | 7.666 | 13.602 | 2.062 | 2.5222 | 71.617 | 54.139 |
| AMNH | 187788 | Eurycea lucifuga | 10.953 | 14.363 | 2.188 | 9.298 | 15.391 | 3.256 | 2.166 | 71.541 | 66.2 |
| AMNH | 187789 | Eurycea lucifuga | 8.032 | 13.29 | 1.665 | 6.528 | 15.495 | 2.684 | 1.854 | 52.427 | 51.987 |
| AMNH | 11865 | Eurycea multiplicata | 4.354 | 6.491 | 1.02 | 5.009 | 6.727 | 1.092 | 0.516 | 29.727 | 45.457 |
| AMNH | 32100 | Eurycea multiplicata | 4.78 | 6.747 | 1.143 | 4.495 | 7.688 | 0.936 | 0.679 | 22.446 | 33.738 |
| AMNH | 40358 | Eurycea multiplicata | 8.954 | 11.331 | 2.207 | 10.124 | 14.946 | 3.071 | 1.713 | 87.019 | 55.592 |
| AMNH | 40360 | Eurycea multiplicata | 5.039 | 5.629 | 0.902 | 3.741 | 7.338 | 1.49 | 0.567 | 37.834 | 39.348 |
| AMNH | 40361 | Eurycea multiplicata | 5.32 | 6.945 | 1.294 | 5.286 | 9.274 | 1.653 | 0.867 | 32.81 | 48.767 |
| AMNH | 40362 | Eurycea multiplicata | 5.476 | 6.584 | 0.949 | 4.613 | 8.236 | 1.358 | 0.701 | 23.828 | 42.503 |
| AMNH | 40363 | Eurycea multiplicata | 5.174 | 5.024 | 1.277 | 5.595 | 7.978 | 1.582 | 0.787 | 38.617 | 39.142 |
| AMNH | 52461 | Eurycea multiplicata | 4.014 | 4.943 | 0.778 | 4.127 | 6.104 | 1.146 | 0.919 | 38.646 | 34.283 |
| AMNH | 52462 | Eurycea multiplicata | 4.251 | 5.217 | 0.928 | 4.836 | 5.651 | 1.083 | 0.845 | 25.241 | 29.515 |
| AMNH | 59813 | Eurycea multiplicata | 5.724 | 7.197 | 1.209 | 6.445 | 8.046 | 1.595 | 1.533 | 43.672 | 42.993 |
| AMNH | 143651 | Eurycea multiplicata | 6.275 | 7.502 | 1.252 | 5.899 | 9.353 | 1.604 | 1.014 | 36.441 | 43.965 |
| AMNH | 151182 | Eurycea multiplicata | 6.087 | 4.948 | 1.119 | 5.605 | 6.609 | 1.538 | 0.724 | 31.773 | 35.056 |
| AMNH | 151191 | Eurycea multiplicata | 4.402 | 4.435 | 0.657 | 4.537 | 5.161 | 0.875 | 0.692 | 20.003 | 28.072 |
| AMNH | 151197 | Eurycea multiplicata | 4.612 | 3.678 | 0.78 | 3.555 | 4.343 | 0.714 | 0.512 | 18.615 | 24.05 |
| AMNH | 151199 | Eurycea multiplicata | 4.553 | 6.243 | 1.169 | 4.155 | 6.226 | 1.143 | 0.563 | 29.565 | 37.328 |
| AMNH | 151200 | Eurycea multiplicata | 5.549 | 7.118 | 1.153 | 5.155 | 7.411 | 1.408 | 0.709 | 43.643 | 35.579 |
| AMNH | 151201 | Eurycea multiplicata | 4.659 | 5.967 | 1.149 | 3.974 | 7.376 | 1.286 | 0.677 | 32.258 | 33.998 |
| AMNH | 151202 | Eurycea multiplicata | 4.561 | 5.988 | 0.73 | 3.641 | 6.972 | 1.056 | 0.856 | 19.928 | 25.097 |
| AMNH | 182174 | Eurycea multiplicata | 5.815 | 6.828 | 1.34 | 6.09 | 7.238 | 1.456 | 0.657 | 42.875 | 41.026 |
| AMNH | 182175 | Eurycea multiplicata | 5.159 | 5.288 | 1.118 | 5.799 | 5.862 | 1.594 | 0.868 | 40.177 | 39.775 |
| AMNH | 60789 | Eurycea nana | 2.986 | 3.264 | 0.48 | 2.347 | 5.164 | 0.821 | 0.596 | 19.642 | 27.167 |
| AMNH | 108768 | Eurycea nana | 3.882 | 5.843 | 0.601 | 4.271 | 5.877 | 0.902 | 0.664 | 19.53 | 29.33 |
| AMNH | 108769 | Eurycea nana | 3.368 | 3.89 | 0.594 | 3.537 | 3.359 | 0.629 | 0.52 | 20.229 | 24.692 |
| AMNH | 108771 | Eurycea nana | 3.638 | 4.533 | 0.602 | 2.824 | 4.949 | 0.613 | 0.424 | 20.188 | 22.547 |
| AMNH | 108772 | Eurycea nana | 3.417 | 5.002 | 0.516 | 2.73 | 4.618 | 0.626 | 0.34 | 20.988 | 27.376 |
| AMNH | 108773 | Eurycea nana | 3.723 | 5.191 | 0.381 | 2.287 | 6.105 | 0.558 | 0.774 | 24.302 | 28.077 |
| AMNH | 108774 | Eurycea nana | 3.613 | 4.718 | 0.586 | 3.562 | 4.78 | 0.72 | 0.809 | 20.604 | 25.641 |
| AMNH | 108776 | Eurycea nana | 3.617 | 4.129 | 0.503 | 2.773 | 4.515 | 0.803 | 0.566 | 17.902 | 22.201 |
| AMNH | 108778 | Eurycea nana | 3.122 | 3.718 | 0.376 | 2.476 | 4.01 | 0.493 | 0.438 | 18.686 | 25.354 |
| AMNH | 108779 | Eurycea nana | 3.235 | 3.815 | 0.467 | 2.206 | 4.426 | 0.59 | 0.571 | 15.101 | 19.991 |
| AMNH | 182176 | Eurycea nana | 3.32 | 3.12 | 0.489 | 3.161 | 3.91 | 0.64 | 0.35 | 23.853 | 31.601 |
| AMNH | 182177 | Eurycea nana | 2.971 | 2.73 | 0.336 | 1.935 | 4.549 | 0.397 | 0.401 | 14.66 | 24.445 |
| AMNH | 182178 | Eurycea nana | 2.976 | 2.653 | 0.35 | 2.36 | 3.242 | 0.357 | 0.306 | 16.872 | 23.01 |
| AMNH | 182179 | Eurycea nana | 3.015 | 3.206 | 0.327 | 2.273 | 3.566 | 0.495 | 0.347 | 18.317 | 23.455 |
| NMNH | 545578 | Eurycea nana | 3.942 | 4.365 | 0.671 | 4.111 | 4.805 | 0.663 | 1.18 | 24.67 | 25.87 |
| NMNH | 545579 | Eurycea nana | 3.85 | 4.328 | 0.557 | 3.774 | 4.751 | 0.593 | 0.658 | 23.052 | 29.037 |
| NMNH | 545581 | Eurycea nana | 3.459 | 3.624 | 0.428 | 2.733 | 3.445 | 0.613 | 0.883 | 16.847 | 21.704 |
| NMNH | 545584 | Eurycea nana | 3.849 | 4.198 | 0.726 | 3.413 | 4.944 | 0.763 | 0.859 | 24.114 | 24.876 |
| NMNH | 545585 | Eurycea nana | 3.717 | 4.445 | 0.561 | 2.654 | 4.42 | 0.616 | 1.063 | 16.24 | 25.344 |
| NMNH | 545587 | Eurycea nana | 3.526 | 3.725 | 0.566 | 2.78 | 3.862 | 0.693 | 0.754 | 17.544 | 21.285 |
| NMNH | 545588 | Eurycea nana | 3.455 | 3.387 | 0.408 | 3.009 | 3.648 | 0.458 | 0.55 | 17.417 | 19.928 |
| NMNH | 545589 | Eurycea nana | 3.343 | 3.643 | 0.408 | 3.31 | 4.756 | 0.479 | 0.822 | 19.483 | 19.707 |
| AMNH | 62054 | Eurycea neotenes | 4.413 | 6.355 | 0.791 | 3.876 | 7.014 | 0.89 | 0.805 | 27.319 | 31.503 |
| AMNH | 62057 | Eurycea neotenes | 4.267 | 5.273 | 0.855 | 3.45 | 5.815 | 0.9 | 0.478 | 23.643 | 32.456 |
| AMNH | 62058 | Eurycea neotenes | 3.797 | 4.597 | 0.87 | 3.011 | 5.412 | 0.895 | 0.698 | 16.88 | 23.318 |
| AMNH | 188124 | Eurycea neotenes | 4.441 | 5.759 | 0.959 | 5.434 | 6.721 | 0.994 | 0.618 | 31.609 | 35.816 |
| AMNH | 188126 | Eurycea neotenes | 4.474 | 4.929 | 0.729 | 3.432 | 5.57 | 0.808 | 0.642 | 22.39 | 27.117 |
| AMNH | 188127 | Eurycea neotenes | 5.318 | 6.163 | 0.934 | 4.463 | 6.899 | 1.032 | 0.675 | 26.234 | 36.746 |
| AMNH | 188128 | Eurycea neotenes | 4.612 | 5.763 | 0.656 | 3.941 | 5.792 | 1.034 | 0.536 | 26.781 | 31.19 |
| AMNH | 188129 | Eurycea neotenes | 4.174 | 5.522 | 0.627 | 4.139 | 6.166 | 0.696 | 0.581 | 29.217 | 32.044 |
| AMNH | 188130 | Eurycea neotenes | 4.792 | 5.857 | 1.021 | 4.846 | 4.551 | 1.163 | 0.809 | 24.549 | 31.537 |
| AMNH | 188133 | Eurycea neotenes | 4.751 | 5.524 | 0.699 | 4.385 | 6.061 | 1.081 | 0.508 | 28.723 | 34.204 |
| AMNH | 188134 | Eurycea neotenes | 3.666 | 3.985 | 0.45 | 3.959 | 5.51 | 0.956 | 0.628 | 18.375 | 26.718 |
| AMNH | 188137 | Eurycea neotenes | 4.316 | 5.227 | 0.861 | 4.166 | 5.205 | 0.65 | 0.775 | 24.528 | 33.44 |
| AMNH | 188138 | Eurycea neotenes | 4.296 | 4.019 | 0.598 | 3.799 | 3.672 | 0.637 | 0.549 | 20.881 | 26.094 |
| AMNH | 188139 | Eurycea neotenes | 5.276 | 5.353 | 0.834 | 4.311 | 6.129 | 0.933 | 0.462 | 25.186 | 32.159 |
| AMNH | 188145 | Eurycea neotenes | 5.43 | 5.677 | 0.674 | 4.332 | 5.998 | 0.774 | 0.504 | 25.324 | 35.402 |
| AMNH | 188150 | Eurycea neotenes | 4.429 | 3.347 | 0.664 | 4.815 | 4.675 | 0.9 | 0.47 | 24.182 | 29.331 |
| AMNH | 188151 | Eurycea neotenes | 4.714 | 4.733 | 0.752 | 4.325 | 5.967 | 0.773 | 0.49 | 23.555 | 29.318 |
| AMNH | 188172 | Eurycea neotenes | 3.738 | 3.956 | 0.569 | 3.424 | 4.509 | 0.842 | 0.345 | 20.232 | 25.652 |
| AMNH | 188198 | Eurycea neotenes | 4.991 | 5.099 | 0.961 | 6.135 | 7.017 | 0.866 | 0.954 | 25.301 | 36.295 |
| AMNH | 188199 | Eurycea neotenes | 5.853 | 6.264 | 0.993 | 5.131 | 6.411 | 1.2 | 0.996 | 26.958 | 35.955 |
| AMNH | 188200 | Eurycea pterophila | 5.911 | 5.176 | 1.194 | 5.35 | 4.985 | 1.358 | 0.782 | 28.249 | 34.534 |
| AMNH | 44336 | Eurycea quadridigitata | 4.828 | 5.844 | 0.803 | 5.204 | 7.532 | 1.128 | 0.926 | 51.089 | 35.927 |
| AMNH | 44337 | Eurycea quadridigitata | 3.627 | 4.051 | 0.963 | 4.226 | 6.384 | 1.167 | 0.5 | 29.046 | 29.681 |
| AMNH | 53905 | Eurycea quadridigitata | 3.848 | 5.036 | 0.55 | 3.78 | 6.018 | 1.036 | 0.603 | 46.393 | 30.356 |
| AMNH | 72593 | Eurycea quadridigitata | 3.817 | 5.975 | 0.745 | 4.101 | 7.549 | 1.105 | 0.826 | 36.709 | 28.646 |
| AMNH | 89818 | Eurycea quadridigitata | 3.421 | 4.879 | 0.533 | 2.91 | 6.565 | 0.807 | 0.881 | 31.827 | 26.088 |
| AMNH | 89821 | Eurycea quadridigitata | 4.334 | 5.826 | 0.968 | 5.19 | 6.495 | 1.246 | 0.772 | 48.685 | 33.625 |
| AMNH | 89822 | Eurycea quadridigitata | 4.217 | 6.459 | 0.866 | 4.352 | 7.419 | 1.149 | 0.925 | 53.357 | 34.102 |
| AMNH | 89828 | Eurycea quadridigitata | 4.04 | 5.009 | 0.882 | 4.944 | 6.397 | 1.151 | 0.806 | 41.638 | 29.982 |
| AMNH | 89834 | Eurycea quadridigitata | 3.985 | 5.4 | 0.698 | 4.061 | 7.704 | 1.092 | 0.848 | 47.968 | 32.708 |
| AMNH | 89835 | Eurycea quadridigitata | 3.24 | 4.664 | 0.735 | 3.839 | 5.792 | 1.086 | 0.833 | 36.601 | 28.894 |
| AMNH | 93055 | Eurycea quadridigitata | 4.078 | 3.639 | 0.98 | 3.969 | 6.284 | 1.13 | 0.555 | 37.025 | 27.249 |
| AMNH | 93058 | Eurycea quadridigitata | 3.697 | 3.735 | 0.859 | 3.466 | 5.011 | 0.847 | 0.33 | 31.966 | 26.554 |
| AMNH | 125819 | Eurycea quadridigitata | 4.986 | 5.829 | 0.911 | 5.222 | 6.839 | 1.152 | 0.799 | 49.504 | 30.402 |
| AMNH | 143803 | Eurycea quadridigitata | 4.119 | 5.658 | 0.944 | 3.864 | 6.729 | 1.314 | 0.778 | 40.532 | 28.052 |
| AMNH | 172401 | Eurycea quadridigitata | 4.296 | 6.037 | 0.928 | 4.229 | 7.842 | 1.166 | 0.909 | 45.762 | 35.188 |
| AMNH | 172404 | Eurycea quadridigitata | 4.418 | 6.987 | 0.85 | 4.625 | 7.9 | 1.13 | 1.199 | 39.295 | 37.502 |
| AMNH | 182183 | Eurycea quadridigitata | 3.76 | 4.107 | 1.03 | 4.726 | 6.88 | 1.159 | 0.785 | 40.634 | 31.121 |
| AMNH | 188213 | Eurycea quadridigitata | 4.443 | 6.428 | 0.945 | 4.113 | 6.708 | 1.218 | 0.926 | 41.036 | 28.112 |
| AMNH | 188214 | Eurycea quadridigitata | 4.275 | 5.769 | 0.923 | 3.828 | 6.947 | 1.007 | 0.861 | 41.612 | 29.177 |
| AMNH | 188215 | Eurycea quadridigitata | 4.029 | 5.912 | 0.771 | 4.516 | 7.007 | 0.987 | 1.152 | 40.07 | 27.14 |
| AMNH | 188216 | Eurycea quadridigitata | 4.889 | 6.481 | 1.113 | 5.582 | 7.747 | 1.231 | 0.704 | 44.672 | 34.063 |
| AMNH | 2276 | Eurycea rathbuni | 15.178 | 16.763 | 1.585 | 8.817 | 15.741 | 2.083 | 1.228 | 41.758 | 60.874 |
| AMNH | 2279 | Eurycea rathbuni | 12.573 | 14.845 | 1.436 | 8.167 | 17.606 | 2.811 | 0.998 | 33.12 | 66.526 |
| AMNH | 2281 | Eurycea rathbuni | 13.107 | 13.681 | 1.613 | 7.797 | 14.035 | 1.943 | 0.73 | 25.253 | 61.258 |
| AMNH | 2282 | Eurycea rathbuni | 10.741 | 13.084 | 1.253 | 6.995 | 12.778 | 1.444 | 0.852 | 30.563 | 47.601 |
| AMNH | 2285 | Eurycea rathbuni | 13.017 | 9.768 | 1.288 | 7.297 | 15.52 | 2.5 | 0.755 | 32.738 | 52.65 |
| AMNH | 2288 | Eurycea rathbuni | 9.153 | 10.799 | 1.359 | 6.135 | 8.997 | 1.112 | 1.684 | 23.054 | 39.645 |
| AMNH | 22645 | Eurycea rathbuni | 7.242 | 10.727 | 1.083 | 5.569 | 11.492 | 1.169 | 1.576 | 26.419 | 38.502 |
| AMNH | 22646 | Eurycea rathbuni | 8.963 | 14.441 | 1.592 | 7.132 | 14.458 | 1.799 | 1.591 | 35.454 | 46.041 |
| AMNH | 22647 | Eurycea rathbuni | 10.968 | 18.098 | 1.271 | 9.007 | 12.671 | 1.28 | 0.661 | 38.891 | 43.941 |
| AMNH | 51178 | Eurycea rathbuni | 10.868 | 17.498 | 1.548 | 8.488 | 16.589 | 1.427 | 0.944 | 39.659 | 47.826 |
| AMNH | 58632 | Eurycea rathbuni | 9.435 | 11.762 | 0.6 | 6.466 | 14.029 | 0.654 | 1.396 | 19.612 | 38.21 |
| AMNH | 58633 | Eurycea rathbuni | 7.66 | 12.89 | 0.786 | 5.515 | 15.126 | 1.122 | 0.928 | 30.47 | 39.804 |
| AMNH | 58634 | Eurycea rathbuni | 9.131 | 13.541 | 0.92 | 5.852 | 13.763 | 0.637 | 1.014 | 29.91 | 35.948 |
| AMNH | 62119 | Eurycea rathbuni | 8.709 | 12.498 | 1.096 | 5.096 | 16.424 | 1.462 | 0.724 | 33.19 | 46.13 |
| AMNH | 155730 | Eurycea rathbuni | 10.664 | 18.214 | 1.385 | 9.056 | 18.058 | 1.368 | 0.944 | 40.94 | 41.93 |
| NMNH | 37051 | Eurycea spelaea | 9.574 | 12.189 | 2.591 | 9.017 | 16.097 | 3.098 | 1.72 | 52.605 | 61.962 |
| NMNH | 37052 | Eurycea spelaea | 9.625 | 10.293 | 2.116 | 8.231 | 9.96 | 2.682 | 1.687 | 50.168 | 56.719 |
| NMNH | 37053 | Eurycea spelaea | 6.453 | 6.154 | 1.587 | 7.301 | 8.96 | 1.541 | 1.02 | 32.217 | 37.626 |
| NMNH | 38787 | Eurycea spelaea | 9.179 | 10.222 | 2.434 | 10.263 | 11.7 | 2.648 | 1.74 | 52.528 | 55.343 |
| NMNH | 38788 | Eurycea spelaea | 9.66 | 9.351 | 2.696 | 9.217 | 15.377 | 2.141 | 1.861 | 62.835 | 59.135 |
| NMNH | 38789 | Eurycea spelaea | 7.831 | 10.05 | 1.966 | 8.129 | 9.351 | 2.558 | 1.413 | 58.32 | 47.7 |
| NMNH | 54326 | Eurycea spelaea | 9.686 | 12.845 | 2.832 | 9.181 | 11.203 | 3.326 | 2.12 | 50.602 | 61.288 |
| NMNH | 57327 | Eurycea spelaea | 8.797 | 11.938 | 2.456 | 9.19 | 12.274 | 3.03 | 1.83 | 56.315 | 61.181 |
| NMNH | 57332 | Eurycea spelaea | 7.277 | 7.659 | 1.93 | 6.726 | 9.963 | 2.455 | 1.31 | 47.23 | 50.025 |
| NMNH | 153780 | Eurycea tridentifera | 7.174 | 10.33 | 0.798 | 4.793 | 10.786 | 1.067 | 1.797 | 23.801 | 22.868 |
| NMNH | 153781 | Eurycea tridentifera | 6.16 | 6.947 | 0.54 | 3.555 | 7.417 | 0.904 | 1.022 | 27.779 | 29.677 |
| NMNH | 153782 | Eurycea tridentifera | 5.818 | 6.725 | 0.476 | 3.271 | 6.128 | 0.846 | 0.861 | 19.396 | 23.487 |
| NMNH | 153783 | Eurycea tridentifera | 4.693 | 5.917 | 0.558 | 2.983 | 7.277 | 0.899 | 0.933 | 21.884 | 22.235 |
| NMNH | 153784 | Eurycea tridentifera | 4.405 | 6.261 | 0.511 | 2.918 | 6.582 | 0.603 | 1.1 | 18.524 | 21.521 |
| NMNH | 153785 | Eurycea tridentifera | 4.212 | 5.359 | 0.482 | 2.373 | 6.397 | 0.495 | 0.672 | 18.813 | 18.997 |
| AMNH | 62060 | Eurycea tynerensis | 3.272 | 3.587 | 0.579 | 2.901 | 4.211 | 0.858 | 0.384 | 24.989 | 29.724 |
| AMNH | 62061 | Eurycea tynerensis | 3.972 | 5.091 | 0.717 | 3.054 | 5.646 | 0.708 | 0.745 | 26.345 | 28.529 |
| AMNH | 182184 | Eurycea tynerensis | 3.194 | 3.518 | 0.576 | 3.44 | 4.149 | 0.508 | 0.323 | 28.292 | 29.615 |
| AMNH | 80090 | Eurycea wallacei | 3.289 | 4.603 | 0.386 | 1.848 | 5.433 | 0.394 | 0.449 | 13.785 | 19.953 |
| AMNH | 80091 | Eurycea wallacei | 4.449 | 6.423 | 0.506 | 2.566 | 6.109 | 0.647 | 0.418 | 17.967 | 23.595 |
| AMNH | 172387 | Eurycea wallacei | 3.649 | 4.355 | 0.588 | 2.55 | 4.558 | 0.979 | 0.415 | 13.494 | 18.467 |
| AMNH | 182197 | Eurycea wallacei | 3.494 | 5.107 | 0.396 | 2.437 | 4.743 | 0.521 | 0.635 | 13.077 | 17.619 |
| AMNH | 99654 | Eurycea wilderae | 6.685 | 8.435 | 1.52 | 5.64 | 9.61 | 1.811 | 1.225 | 51.368 | 39.189 |
| AMNH | 99655 | Eurycea wilderae | 6.076 | 8.215 | 1.276 | 5.57 | 9.741 | 1.645 | 1.26 | 53.578 | 40.35 |
| AMNH | 99657 | Eurycea wilderae | 6.238 | 7.245 | 1.514 | 5.413 | 7.949 | 1.674 | 1.747 | 42.287 | 36.512 |
| AMNH | 99658 | Eurycea wilderae | 5.77 | 7.604 | 1.446 | 4.929 | 8.422 | 1.876 | 1.226 | 50.049 | 38.119 |
| AMNH | 99659 | Eurycea wilderae | 6.674 | 7.939 | 1.486 | 6.059 | 9.32 | 2.046 | 0.986 | 55.488 | 44.332 |
| AMNH | 99660 | Eurycea wilderae | 5.948 | 8.373 | 1.423 | 5.155 | 10.014 | 1.602 | 1.269 | 53.004 | 40.795 |
| AMNH | 115827 | Eurycea wilderae | 4.737 | 6.677 | 1.064 | 5.266 | 10.054 | 1.49 | 1.282 | 50.934 | 37.154 |
| AMNH | 115829 | Eurycea wilderae | 5.153 | 7.902 | 1.122 | 5.379 | 10.438 | 1.456 | 1.369 | 58.034 | 39.118 |
| AMNH | 115830 | Eurycea wilderae | 5.653 | 8.4 | 1.168 | 6.114 | 11.581 | 1.726 | 1.581 | 65.387 | 42.22 |
| AMNH | 115833 | Eurycea wilderae | 6.095 | 7.743 | 1.26 | 6.179 | 10.168 | 1.925 | 1.335 | 58.364 | 42.062 |
| AMNH | 115834 | Eurycea wilderae | 5.487 | 8.646 | 1.318 | 5.864 | 10.609 | 1.698 | 1.224 | 56.287 | 39.632 |
| AMNH | 115835 | Eurycea wilderae | 5.054 | 8.263 | 1.179 | 5.253 | 10.484 | 1.56 | 1.329 | 55.29 | 36.557 |
| AMNH | 115836 | Eurycea wilderae | 4.917 | 6.858 | 1.296 | 4.718 | 10.181 | 1.461 | 1.131 | 50.933 | 35.509 |
| AMNH | 127036 | Eurycea wilderae | 4.34 | 6.101 | 0.957 | 4.199 | 6.196 | 1.233 | 0.845 | 38.961 | 32.577 |
| AMNH | 127039 | Eurycea wilderae | 4.167 | 5.667 | 0.895 | 4.281 | 7.187 | 1.283 | 0.885 | 38.919 | 32.419 |
| AMNH | 155790 | Eurycea wilderae | 4.81 | 7.12 | 1.019 | 4.852 | 9.405 | 1.196 | 0.863 | 30.622 | 32.061 |
| AMNH | 155791 | Eurycea wilderae | 4.677 | 7.344 | 0.95 | 4.502 | 8.563 | 1.416 | 1.084 | 37.525 | 43.436 |
| AMNH | 171738 | Eurycea wilderae | 5.59 | 7.081 | 1.242 | 5.084 | 8.506 | 1.307 | 1.323 | 26.146 | 44.497 |
| AMNH | 171739 | Eurycea wilderae | 4.971 | 5.148 | 1.103 | 4.63 | 6.714 | 1.226 | 0.564 | 38.34 | 37.97 |
| AMNH | 172392 | Eurycea wilderae | 5.701 | 9.065 | 1.164 | 6.381 | 8.876 | 1.437 | 1.237 | 33.108 | 39.246 |
| AMNH | 95353 | Gyrinophilus porphyriticus | 9.207 | 8.174 | 2.01 | 10.051 | 8.367 | 2.447 | 1.54 | 40.395 | 55.099 |
| AMNH | 137525 | Gyrinophilus porphyriticus | 13.079 | 15.153 | 3.378 | 12.838 | 16.849 | 3.367 | 1.609 | 41.484 | 93.658 |
| AMNH | 137533 | Gyrinophilus porphyriticus | 10.641 | 9.615 | 2.438 | 9.989 | 10.624 | 2.641 | 1.263 | 31 | 62.48 |
| AMNH | 137538 | Gyrinophilus porphyriticus | 12.043 | 9.944 | 2.701 | 11.31 | 14.977 | 3.082 | 1.758 | 42.143 | 70.606 |
| AMNH | 137590 | Gyrinophilus porphyriticus | 10.455 | 9.398 | 2.608 | 9.724 | 9.797 | 2.481 | 1.136 | 40.265 | 57.712 |
| AMNH | 137591 | Gyrinophilus porphyriticus | 10.137 | 9.323 | 2.077 | 10.24 | 11.572 | 2.71 | 1.124 | 39.248 | 56.742 |
| AMNH | 137593 | Gyrinophilus porphyriticus | 9.552 | 9.827 | 2.517 | 10.382 | 9.118 | 2.533 | 0.998 | 42.651 | 58.975 |
| AMNH | 137631 | Gyrinophilus porphyriticus | 12.852 | 11.205 | 3.073 | 11.496 | 16.193 | 3.885 | 2.194 | 45.029 | 78.49 |
| AMNH | 137632 | Gyrinophilus porphyriticus | 10.887 | 13.713 | 2.612 | 10.309 | 15.807 | 3.199 | 1.705 | 38.977 | 75.936 |
| AMNH | 137653 | Gyrinophilus porphyriticus | 17.023 | 17.691 | 4.719 | 18.167 | 21.134 | 5.143 | 2.991 | 72.221 | 119.842 |
| AMNH | 137654 | Gyrinophilus porphyriticus | 16.159 | 18.13 | 3.91 | 17.058 | 21.519 | 5.219 | 2.795 | 76.475 | 113.408 |
| AMNH | 157716 | Gyrinophilus porphyriticus | 13.842 | 16.283 | 3.578 | 17.532 | 19.846 | 3.698 | 2.948 | 87.135 | 85.935 |
| AMNH | 169622 | Gyrinophilus porphyriticus | 24.362 | 11.092 | 3.201 | 12.077 | 13.516 | 3.345 | 2.658 | 62.303 | 77.954 |
| AMNH | 170907 | Gyrinophilus porphyriticus | 8.679 | 9.963 | 2.313 | 9.908 | 13.957 | 3.683 | 2.948 | 46.11 | 68.947 |
| AMNH | 182194 | Gyrinophilus porphyriticus | 10.777 | 12.333 | 2.01 | 10.443 | 18.434 | 2.787 | 1.361 | 55.287 | 77.817 |
| AMNH | 190416 | Gyrinophilus porphyriticus | 14.652 | 12.795 | 3.46 | 15.776 | 18.803 | 4.277 | 4.27 | 45.85 | 106.782 |
| NMNH | 285091 | Hydromantes brunus | 5.902 | 10.953 | 1.13 | 4.501 | 10.857 | 1.397 | 1.667 | 22.557 | 31.64 |
| NMNH | 285093 | Hydromantes brunus | 5.345 | 6.743 | 0.938 | 5.22 | 6.994 | 1.39 | 1.205 | 17.264 | 23.634 |
| NMNH | 285094 | Hydromantes brunus | 5.501 | 6.865 | 0.915 | 5.108 | 6.84 | 1.193 | 1.401 | 17.711 | 22.853 |
| NMNH | 285097 | Hydromantes brunus | 4.432 | 5.9 | 0.667 | 3.315 | 6.102 | 1.494 | 0.985 | 15.192 | 19.85 |
| NMNH | 321295 | Hydromantes brunus | 11.258 | 14.574 | 2.459 | 9.039 | 16.23 | 3.095 | 2.713 | 49.119 | 55.379 |
| NMNH | 545723 | Hydromantes brunus | 7.445 | 9.051 | 1.204 | 5.223 | 10.578 | 1.519 | 1.53 | 21.744 | 30.875 |
| NMNH | 58732 | Hydromantes genei | 7.205 | 9.129 | 1.66 | 5.728 | 9.547 | 1.535 | 1.031 | 22.551 | 33.639 |
| NMNH | 58733 | Hydromantes genei | 4.408 | 4.93 | 0.908 | 3.277 | 5.795 | 0.951 | 0.546 | 11.897 | 18.331 |
| NMNH | 93878 | Hydromantes genei | 10.681 | 15.11 | 2.485 | 10.033 | 18.979 | 4.292 | 2.169 | 50.849 | 58.325 |
| NMNH | 93880 | Hydromantes genei | 10.462 | 16.68 | 2.365 | 8.494 | 22.756 | 2.449 | 2.651 | 49.441 | 59.173 |
| NMNH | 100925 | Hydromantes genei | 10.072 | 19.464 | 2.04 | 7.631 | 17.777 | 2.499 | 2.627 | 61.391 | 41.524 |
| NMNH | 100930 | Hydromantes genei | 10.379 | 15.683 | 1.836 | 8.09 | 18.378 | 2.704 | 2.574 | 48.151 | 53.573 |
| NMNH | 100932 | Hydromantes genei | 9.114 | 16.772 | 1.778 | 8.006 | 14.462 | 2.667 | 1.904 | 37.051 | 52.767 |
| AMNH | 23485 | Hydromantes italicus | 8.681 | 12.366 | 2.01 | 7.472 | 12.389 | 2.325 | 2.182 | 27.145 | 52.033 |
| AMNH | 23645 | Hydromantes italicus | 9.448 | 13.842 | 1.837 | 7.541 | 10.626 | 2.605 | 1.75 | 32.263 | 50.672 |
| AMNH | 24873 | Hydromantes italicus | 8.66 | 10.012 | 2.555 | 7.499 | 10.989 | 1.635 | 1.487 | 34.262 | 47.057 |
| AMNH | 24874 | Hydromantes italicus | 9.674 | 15.047 | 2.506 | 8.552 | 13.641 | 2.21 | 2.905 | 41.339 | 53.212 |
| AMNH | 34631 | Hydromantes italicus | 9.512 | 13.023 | 2.376 | 8.81 | 12.227 | 2.498 | 3.483 | 32.732 | 51.687 |
| AMNH | 34632 | Hydromantes italicus | 8.82 | 15.057 | 2.071 | 7.072 | 16.549 | 1.992 | 1.547 | 37.729 | 54.756 |
| AMNH | 34633 | Hydromantes italicus | 9.568 | 14.473 | 2.353 | 7.432 | 15.293 | 3.36 | 1.627 | 38.346 | 55.567 |
| AMNH | 52692 | Hydromantes italicus | 9.453 | 11.126 | 1.826 | 7.426 | 11.926 | 2.327 | 1.757 | 34.411 | 49.702 |
| AMNH | 54694 | Hydromantes italicus | 10.526 | 18.021 | 2.081 | 10.356 | 17.261 | 2.54 | 1.779 | 33.898 | 65.84 |
| AMNH | 54695 | Hydromantes italicus | 10.329 | 14.518 | 2.081 | 8.642 | 14.167 | 3.415 | 1.593 | 38.649 | 59.431 |
| AMNH | 54696 | Hydromantes italicus | 11.581 | 16.005 | 2.446 | 9.39 | 15.088 | 3.116 | 1.311 | 45.963 | 62.773 |
| AMNH | 65109 | Hydromantes italicus | 9.903 | 13.373 | 2.091 | 7.359 | 14.133 | 2.975 | 1.325 | 35.788 | 58.492 |
| AMNH | 65110 | Hydromantes italicus | 9.685 | 10.16 | 1.946 | 7.604 | 14.598 | 2.421 | 1.193 | 32.221 | 55.705 |
| AMNH | 65111 | Hydromantes italicus | 9.043 | 14.669 | 1.826 | 6.554 | 15.533 | 2.071 | 1.375 | 39.774 | 59.958 |
| AMNH | 65112 | Hydromantes italicus | 6.749 | 8.192 | 1.145 | 4.972 | 10.269 | 1.448 | 1.042 | 23.099 | 34.535 |
| AMNH | 149539 | Hydromantes italicus | 8.539 | 10.594 | 1.441 | 6.36 | 12.542 | 1.761 | 1.643 | 28.134 | 51.758 |
| AMNH | 149540 | Hydromantes italicus | 9.067 | 12.844 | 1.662 | 7.85 | 12.903 | 2.338 | 1.863 | 32.22 | 50.977 |
| AMNH | 149541 | Hydromantes italicus | 10.479 | 14.251 | 1.94 | 8.703 | 14.55 | 2.22 | 1.45 | 42.806 | 58.289 |
| AMNH | 149542 | Hydromantes italicus | 11.107 | 14.12 | 2.255 | 9.779 | 14.971 | 3.408 | 1.104 | 35.204 | 59.952 |
| AMNH | 149543 | Hydromantes italicus | 9.818 | 13.192 | 2.059 | 8.985 | 13.341 | 2.82 | 1.473 | 40.591 | 56.153 |
| AMNH | 53808 | Hydromantes platycephalus | 8.704 | 12.358 | 1.823 | 8.947 | 15.483 | 2.433 | 1.863 | 35.84 | 56.365 |
| AMNH | 53809 | Hydromantes platycephalus | 8.541 | 11.476 | 1.554 | 7.626 | 12.031 | 1.659 | 0.984 | 21.746 | 42.696 |
| AMNH | 53810 | Hydromantes platycephalus | 6.939 | 7.574 | 1.294 | 6.722 | 10.169 | 1.586 | 1.044 | 16.498 | 33.235 |
| AMNH | 53811 | Hydromantes platycephalus | 6.556 | 7.825 | 1.1 | 5.754 | 9.765 | 1.828 | 1.083 | 20.158 | 32.934 |
| AMNH | 53812 | Hydromantes platycephalus | 9.477 | 10.711 | 1.816 | 8.55 | 14.447 | 2.272 | 1.168 | 30.359 | 51.079 |
